# AI for bioactive materials: From material design to biological applications

**DOI:** 10.1016/j.bioactmat.2026.04.028

**Published:** 2026-05-14

**Authors:** Jiezhong Shi, Ting Liang, Marcin Heljak, Zhen Qiu, Song Chen, Jiaying Li, Asimina Kazakidi, Wojciech Święszkowski, Bin Li, Wenmiao Shu

**Affiliations:** a SINOPEC Key Laboratory of Research and Application of Medical and Hygienic Materials, SINOPEC Beijing Research Institute of Chemical Industry Co., Ltd., Beijing, 100013, PR China; b Department of Biomedical Engineering, University of Strathclyde, Glasgow, G4 0NW, UK; c Medical 3D Printing Center, Orthopedic Institute, Department of Orthopedic Surgery, The First Affiliated Hospital, Biomedical Basic Research Center (BBRC) of Jiangsu Province, MOE Key Laboratory of Geriatric Diseases and Immunology, Suzhou Medical College, Soochow University, Suzhou, Jiangsu, 215000, PR China; d Warsaw University of Technology, Faculty of Materials Science and Engineering, ul. Wołoska 141, 02-507, Warsaw, Poland; e Collaborative Innovation Center of Hematology, Soochow University, Suzhou, Jiangsu, 215000, PR China

**Keywords:** Bioactive materials, Artificial intelligence, Machine learning, Property prediction, Material design

## Abstract

Bioactive materials are engineered to actively interact with biological systems and induce favorable cellular and tissue responses. The development of bioactive materials is inherently challenging due to the complexity of the biological processes that they participate in. Traditional high-throughput platforms and computational simulations are often insufficient to address the multidimensional requirements. Artificial intelligence (AI) provides powerful tools to address these challenges. By utilizing data-driven models, AI can reveal non-obvious relationships within experimental datasets, enable prediction of material behaviors, and guide rational design of new materials. In this review, we provide a systematic overview of recent advances in AI for bioactive materials. Firstly, the classification of bioactive materials is summarized, including bioactive metals, bioactive ceramics, bioactive polymers and bioactive composites. Then, current AI tools employed in bioactive materials are presented according to various classification methods. Next, the process-oriented applications of AI in bioactive materials are introduced, such as material design, fabrication optimization, property prediction, in vitro assessments, and in vivo assessments. Finally, the key challenges and future opportunities in this field are discussed comprehensively.

## Highlights

• The classification of bioactive materials is discussed including metals, ceramics, polymers and composites.
• Recent AI tools and frameworks employed in bioactive material research are systematically summarized.
• AI applications from design and fabrication to property prediction and biological analysis are highlighted.
• The challenges and opportunities in AI-driven innovation in bioactive materials are discussed.

## Graphical abstract

**Figure:**
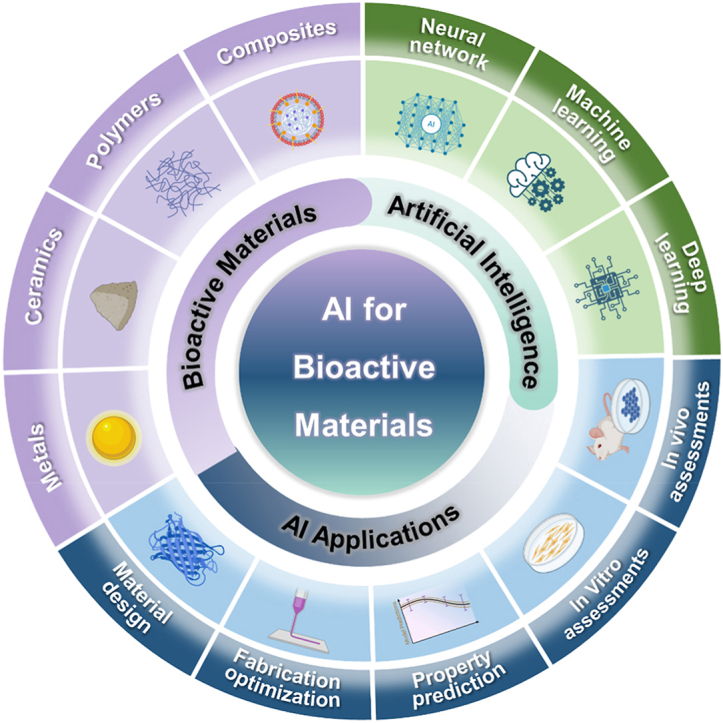

## 1 Introduction

Biomaterials [1], in a general sense, encompass all materials intended for application within biological systems. These materials can be derived from diverse sources and are typically categorized into metals, ceramics, and polymers [2]. Biomaterials are widely used in a wide range of biomedical applications, such as tissue scaffolds, wound healing, drug delivery and disease diagnosis, etc [3,4]. Given the intricate and dynamic nature of physiological environments, the development of biomaterials usually needs extensive experimental studies. Beyond meeting mechanical requirements, biomaterials have to exhibit excellent biocompatibility to minimize adverse biological reactions [5].

Within the broad category of biomaterials, bioactive materials represent a specialized subset that are designed not only to coexist with biological systems, but also to interact with biological systems to induce beneficial responses, including promoting cell interaction, facilitating tissue regeneration and modulating immune activity [[6], [7], [8]]. Different from biomaterials, bioactive materials include certain metals such as magnesium [9], which offers both degradability and osteoconductivity, and bioactive ceramics like calcium phosphate [10], known for their bone-bonding ability. In addition, natural polymers and synthetic polymers with bioactive moieties are also involved [11]. Since bioactive materials are intended to actively participate in biological processes rather than passively serving as structural supports, their evaluation extends beyond traditional mechanical and biocompatibility assessments. The cell behaviors and cell-material interactions must also be comprehensively studied [12,13]. Particularly for implantable materials, careful consideration of their immunogenicity and degradability is essential to ensure the safe and effective integration within the body [14]. These multifactorial criteria make the research of bioactive materials inherently more complex and interdisciplinary.

In order to address the complexity in the development of bioactive materials, high-throughput platforms have been introduced as an efficient solution to accelerate early-stage screening [15]. However, the development of bioactive materials encompasses a series of interrelated steps, including structure design, material fabrication, property characterization, data analysis, biological evaluation, and immunological assessment, which exceed the current capabilities of high-throughput platforms. Computational simulations offer another valuable approach by predicting material properties and guiding material design, thereby simplifying the development process of bioactive materials [16]. Nevertheless, the large amounts of data and images generated during experiments pose significant analytical challenges, and conventional computational methods often fall short in extracting deep, non-obvious patterns from large datasets. Moreover, computational simulations are generally limited to relatively simple molecular structures. When applied to biomacromolecules like peptides that exhibit extensive sequence variability and structural complexity, current simulation methods remain inadequate.

To overcome these limitations, data-driven approaches, particularly those based on artificial intelligence (AI), are increasingly being integrated into bioactive material research [[16], [17], [18], [19], [20], [21], [22], [23], [24], [25], [26]]. By utilizing abundant experimental data as inputs, AI can uncover hidden patterns, predict material properties, and even propose novel material formulations that might be overlooked by traditional “trial and error” method [25]. The AI tools in bioactive materials primarily rely on machine learning (ML), which can continuously improve their predictive and decision-making accuracy as more experimental data become available. With ongoing advancements in computational power, AI is expected to play a pivotal role in accelerating the discovery, optimization, and clinical translation of next-generation bioactive materials. Although there are already a few reviews on AI for biomaterials [[16], [17], [18], [19], [20], [21], [22], [23], [24], [25], [26]], they primarily concentrate on biomaterials rather than bioactive materials. Bioactive materials inherently involve complex interactions with cells and tissues, generating massive complex data that are difficult to analyze manually or with traditional statistical approaches. AI is particularly well-suited to addressing this challenge, making it an invaluable tool to capture these multifaceted processes, extracting hidden patterns, and guiding rational material design. In addition, the majority of reviews on AI applications in this field tend to classify them by material types while neglecting the development processes, which limits their ability to provide a comprehensive understanding and practical guidance for future research. In contrast, this review narrows the scope to bioactive materials and adopts a unique process-oriented perspective to discuss AI applications, thereby offering a more systematic framework to elucidate current progress, identify critical challenges, and inspire future directions towards the clinical translation of bioactive materials.

**Fig. 1:**
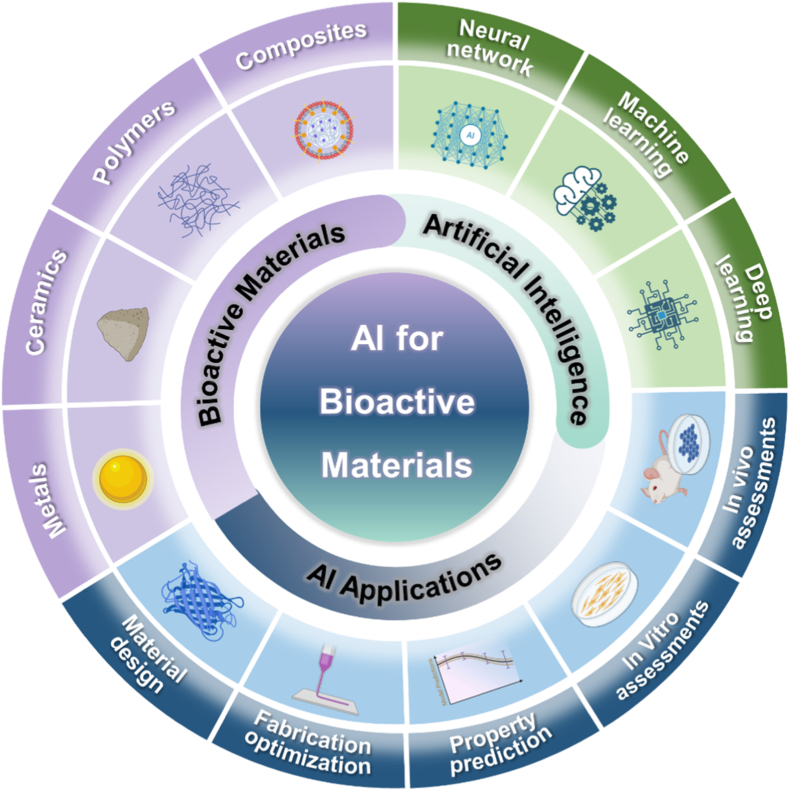
Scheme of bioactive materials, AI tools and the applications of AI in bioactive materials. Bioactive materials include metals, ceramics, polymers and composites. AI tools are mainly based on artificial neural network, machine learning and deep learning. Applications across different stages of material development encompass material design, fabrication optimization, property prediction, in vitro assessments, and in vivo assessments.

In this review, an overview of bioactive materials is firstly provided in Section 2, including bioactive metals, bioactive ceramics, bioactive polymers and bioactive composites. In Section 3, the current AI tools used in bioactive material research are based on different classification methods, with a particular focus on ML and AI-based quantitative structure-activity relationships (QSAR) tools. In Section 4, the applications of AI in bioactive materials across different stages of material development are summarized such as material design, fabrication optimization, property prediction, in vitro assessments, and in vivo assessments (Fig. 1). Finally, the challenges and opportunities in integrating AI into the development of bioactive materials are discussed in Section 5.

## 2 Bioactive materials

**Fig. 2:**
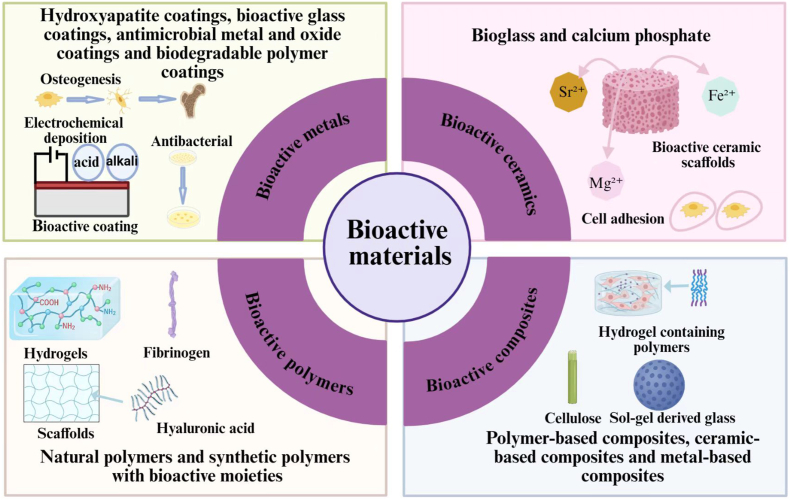
Classification of bioactive materials, including bioactive metals with hydroxyapatite and other coatings, bioactive ceramics including bioglass and calcium phosphate, bioactive polymers including natural polymers and synthetic polymers with bioactive moieties, and bioactive composites including polymer-based composites, ceramic-based composites and metal-based composites.

Bioactive materials are substances that can interact dynamically with biological systems to induce desired physiological responses, such as promoting cell adhesion and proliferation, accelerating tissue repair and regeneration, and regulating immune activation and signaling. Rather than acting solely as passive structural supports, these materials are designed to participate in the biological processes actively in a controlled manner [[27], [28], [29], [30]]. 3D printed porous magnesium metal scaffolds with bioactive coatings can be used for bone repair, capable of enhancing angiogenesis and promoting osteogenesis. Non-degradable titanium alloy implants are commonly used in knee joint replacement. Bioactive glass (BG) can actively regulate the microenvironment for bone regeneration to facilitate bone injury repair. Polylactic acid (PLA) scaffolds can be used for soft tissue regeneration and drug delivery systems, while hydroxyapatite (HA)/polymer composites are widely used in bone tissue engineering [31,32]. This section provides an overview of the commonly used bioactive materials classified according to their chemical compositions, including bioactive metals, ceramics, polymers, and composites (Fig. 2).

### 2.1 Bioactive metals: metals with bioactive coatings

**Fig. 3:**
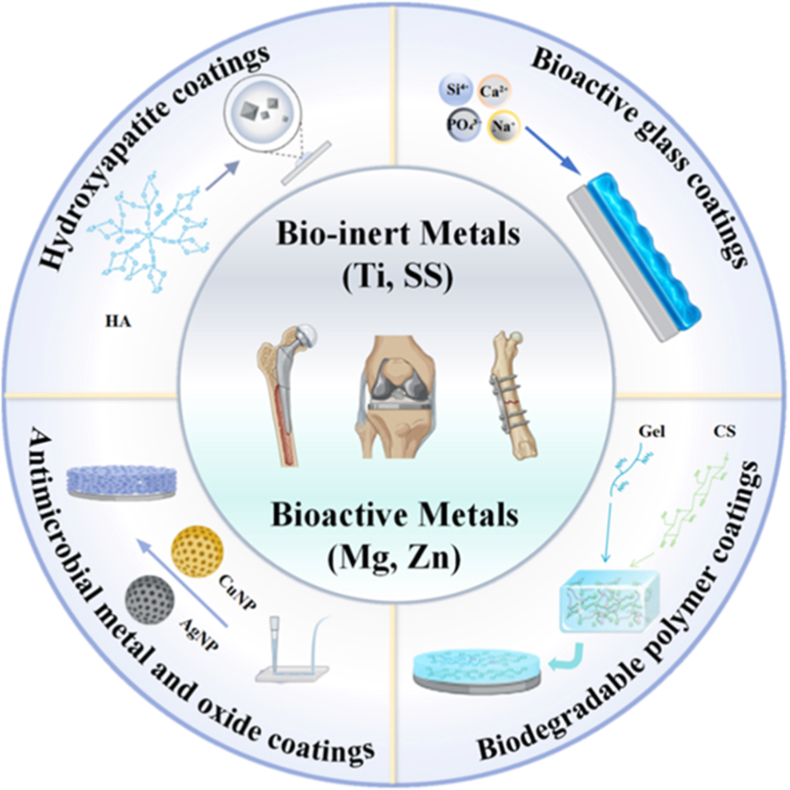
Classification of bioactive metals and their surface coatings. Bioactive metals are divided into bio-inert metals (e.g., Ti and SS) and bioactive metals (e.g., Mg and Zn), with four coating types including hydroxyapatite coatings, bioactive glass coatings, antimicrobial metal and oxide coatings and biodegradable polymer coatings.

Metallic materials such as stainless steel, titanium alloys and magnesium alloys are widely used as implants-especially in orthopedics and dentistry-because of their excellent mechanical properties [33]. The surface coatings of metallic implants are mainly designed based on two different clinical strategies: the permanent stability of bioinert metals and the controlled degradability of bioresorbable metals. For bioinert metals such as titanium or stainless steel, the coatings are primarily used to promote osseointegration and provide protection against wear and ion release. In contrast, for bioresorbable metals such as magnesium or zinc, the coatings play a temporary regulatory role. These coatings are specially designed to synchronize the disappearance of the implant with the timeline of tissue healing, either by delaying rapid corrosion to maintain mechanical integrity or by accelerating slow degradation to ensure complete material absorption (Fig. 3).

Based on their material composition and functional properties, these coatings can be classified into hydroxyapatite coatings, bioactive glass coatings, antimicrobial metal and oxide coatings, biodegradable polymer coatings. In addition, the performance of the coating largely depends on the preparation and modification techniques adopted, mainly including two categories: coating deposition and electrochemical surface modification.

#### 2.1.1 Hydroxyapatite coatings

In the field of orthopedic implant materials, bioceramic coatings with both osteogenic induction and antibacterial functions can be constructed on the surface of metal substrates, so as to achieve long-term stability of implants in orthopedic clinical practice. HA has become the most widely studied ceramic material in this field due to its chemical composition, crystal structure, high consistency with natural bone inorganic phase, outstanding biocompatibility and biological activity [34]. HA coatings can not only be prepared on the surface of titanium alloys and other metals through various processes such as thermal spraying, electrophoretic deposition, and pulsed laser deposition, but also mimic the natural bone microenvironment, significantly promote cell adhesion and proliferation, induce osteoblast differentiation, and accelerate bone tissue formation [35]. HA coatings constructed on the surface of titanium alloys have been shown to improve biocompatibility and cell osteogenic differentiation [36,37]. Dong and co-workers prepared a gradient HA coating on Ti6Al4V by laser engineering net shaping and plasma spray deposition to improve bonding strength, thereby improving bone integration and reducing the risk of implant failure due to loosening or infection [36]. Zhang and co-workers further demonstrated that the expression of osteocalcin, osteogenic differentiation factor and other genes was up-regulated and alkaline phosphatase (ALP) activity was significantly enhanced after the X-type porous Ti6Al4V scaffold was modified with HA coating, which directly confirmed the promoting effect of HA coating on osteogenic differentiation [38]. However, the porous structure of HA is prone to bacterial growth, and it is necessary to enhance the antibacterial properties by incorporating antibacterial elements (e.g., Cu, Ag, Zn) or compound antibiotics, such as Ti-Cu alloy and tobramycin-HA composite coating, which can effectively inhibit infection [[39], [40], [41]]. HA coatings improve corrosion resistance of magnesium implants on magnesium surfaces [42]. With the deepening of nanotechnology and surface modification technology, nano-HA coatings are expected to bring broader application prospects in the field of bone repair.

#### 2.1.2 Bioactive glass coatings

BG can rapidly release active ions such as Si^4+^, Ca^2+^, Na^+^, and PO_4_^3−^ in vivo, induce the in-situ generation of osteoid HA layers, and form chemical bonds with the host bone, thereby significantly accelerating osseointegration and stabilizing the implant for a long time [43,44]. As a coating for metal implants, BG has the functions of early fixation, inhibition of metal ion release and corrosion reduction. Thick/thin coatings can be prepared as needed by various processes such as sol-gel and electrophoretic deposition [45]. Costa and co-workers constructed BG-based coatings on Ti surfaces with the help of plasma electrolytic oxidation (PEO), which exhibited excellent corrosion resistance, high protein adsorption capacity, and excellent biocompatibility [46]. BG coatings can also synergistically regulate cell adhesion, proliferation, differentiation, mineralization, angiogenesis, and anti-inflammatory responses through nanoscale roughening and ion release. In terms of osteogenic performance, Bargavi and co-workers used pulsed laser deposition to construct a Zr-BG composite coating on the surface of titanium, and found that the continuous release of Ca^2+^/Si^4+^ significantly upregulated collagen synthesis and ALP activity in MC3T3-E1 cells, and the mineralized nodule area accounted for 38% after 14 days, which was much higher than that of the pure titanium group (11%), fully verifying its ability to accelerate osteogenic differentiation and matrix mineralization [44]. Rau and co-workers used the sol-gel method to construct a Cu-BG coating on the surface of titanium alloy to achieve Cu^2+^ gradient controlled release, which not only promoted the early differentiation of osteoblasts, but also significantly down-regulated the expression of the inflammatory factor IL-1β [47]. Although BG itself has limited antibacterial activity, it can confer broad-spectrum antibacterial and anti-inflammatory effects by compounding with functional ions such as Ag^+^, Zn^2+^, and Cu^2+^ [[48], [49], [50]]. In summary, BG coatings provide a promising solution for the next generation of metal orthopedic implants with osteogenic, antibacterial and anti-inflammatory properties through micro/nanostructure regulation, elemental doping and multifunctional compounding [51].

#### 2.1.3 Antimicrobial metal and oxide coatings

Antimicrobial metals and their oxide coatings can directly kill pathogenic bacteria on the surface of metal implants by releasing metal ions locally, promote osseointegration, and improve biocompatibility, providing new strategies for infection prevention. Silver nanoparticles (AgNPs) and copper nanoparticles (CuNPs) can anchor on the bacterial cell wall and infiltrate the bacteria, leading to physical changes in the bacterial membrane, such as membrane damage, resulting in leakage of cell contents and bacterial death [52]. AgNPs and CuNPs can interfere with the activity of bacterial DNA replication and metabolic enzymes through the release of Ag^+^ and Cu^2+^, and cause increased oxidative stress in microbial cells [53]. AgNPs also have anti-biofilm activity [54]. AgNPs have good biocompatibility in osteoblast-related cells (osteoblasts, osteoclasts, mesenchymal stem cells (MSCs), etc.), which can improve the activity, adhesion, proliferation and differentiation of these cells. Cu can also promote bone development by catalyzing metabolic processes and play an important role in bone growth and bone mass maintenance in humans [55].

In the field of orthopedic research, AgNP-coated external fixation needles, giant prostheses of the femur or tibia, and AgNPs containing bone cement have been innovated and show a trend of infection inhibition [56,57]. Adriana and co-workers developed a bifunctional peptide that promotes the adhesion of AgNPs and titanium, which has antimicrobial activity against the major bacteria that cause peri-implant infections in titanium-based orthopedic devices [58]. Liu and co-workers found that magnetron sputtering can be used to evenly distribute AgNP coatings on the surface of polyether ether ketone (PEEK) samples [59]. AgNP-coated PEEK implant material has a strong antibacterial and bactericidal effect on *Streptococcus mutans* and *Staphylococcus aureus* in the oral cavity. Aziz and co-workers used AgNPs, CuNPs, and copper oxide nanoparticles synthesized from quercetin or cactus leaf green to physicochemically characterize nanocomposites through Fourier transform infrared spectroscopy (FTIR), X-ray diffraction (XRD), energy dispersive X-ray spectroscopy (EDX), etc., and found their potential in antibacterial, antibiofilm, and antioxidant therapy [60,61]. Khalil and co-workers deposited a double-layer composite coating with PEEK as the first layer and titanium dioxide (TiO_2_) and Cu-doped mesoporous bioactive glass nanoparticles (Cu-MBGNs) as the second layer on austenitic low-carbon stainless steel (316L SS) by electrophoresis deposition, which significantly improved the wear resistance and corrosion resistance of 316L SS, and the coating exhibits strong antibacterial effects against *Staphylococcus aureus* and *Escherichia coli* [62]. 3D bioprinting technology enables the temporospatially controlled distribution of “bioinks” containing cells with regenerative potential, scaffolds doped with metal/ion nanoparticles, and biomacromolecules [63]. Doping AgNPs into scaffolds with antimicrobial activity and biocompatibility properties in musculoskeletal tissues helps to inhibit infection and promote tissue regeneration [64]. Damle and co-workers demonstrated the proliferation and differentiation of temporal filling stromal cells doped with AgNPs, which provides further insights for bone tissue engineering [65]. Xu and co-workers prepared a composite coating composed of HA, chitosan (CS), tannic acid (TA), and Cu^2+^ on the surface of a 3D-printed porous Ti alloy scaffold by electrophoretic deposition method, and found that the composite coating had better antibacterial properties and cytocompatibility and lower cytotoxicity [66]. Antimicrobial metals and their oxides show good antibacterial properties and low infection rates after bone implantation through various mechanisms, and synergistically promote bone regeneration [[67], [68], [69], [70], [71]], and their application to bioactive materials helps optimize their biocompatibility and functional integration to promote clinical translation.

#### 2.1.4 Biodegradable polymer coatings

Biodegradable polymer coatings can provide sustained antibacterial activity for metal implants, promote immune reconstruction, and achieve robust bone integration [72]. By imparting temporal regulation functions to traditional plant materials, they also enable precise interventions in orthopedic defect repair and regeneration processes. After fulfilling their intended purpose, they can decompose within the human body without causing harm to the host. CS is a natural polymer compound with cationic characteristics obtained after deacetylation of chitin, which can be hydrolysed into harmless products by the human enzyme system [73], and is a high-quality material for orthopedic tissue engineering scaffolds, with high biocompatibility, biodegradability, antibacterial properties, excellent mechanical properties, and low immunogenicity [74,75]. Polycaprolactone (PCL) is a widely used synthetic aliphatic polyester with a semi-crystalline structure that is very biodegradable and biocompatible. However, its hydrophobicity limits biological activity, which can be eliminated by binding hydrophilic substances. In addition, it can promote cell adhesion, cell differentiation, and proliferation by binding with CS [76]. Gelatin is a product of collagen hydrolysis and has good biocompatibility. Gelatin activates macrophages, shows high hemostatic effects, and does not cause antigenicity.

Li and co-workers used nano-TiO_2_ modified chitosan/gelatin/aldehyde hyaluronic acid (CS/Gel/AHA) hydrogel, combined with dipping technology, to prepare a coating with good mechanical strength; the released Ca^2+^ acts on the bacterial Bap protein, inhibiting the formation of biofilm, while the released vancomycin kills free bacteria, providing a feasible method for the treatment of chronic osteomyelitis [77]. Azam and co-workers found that Cu-substituted nano-hydroxyapatite (nHA) incorporated into CS/gelatin composite scaffolds provides a highly porous 3D structure with good physicochemical properties, mechanical strength, and can sustainably promote Cu release as well as osteoblast attachment, proliferation, and mineralization [78]. Knigge and co-workers assessed corrosion of coated magnesium using an immersion test in simulated body fluids by spinning on the surface layer of a magnesium implant. The study found that the PCL coating provided effective protection against corrosion erosion, with a significant 75% reduction in corrosion rates [79]. To regulate the degradation rate of AZ31B magnesium alloy and improve surface biocompatibility, Daavari and co-workers produced three different coating systems by PEO: simple PEO, PEO containing multi-walled carbon nanotubes (PEO CNT), and duplex coating containing polycaprolactone top layer (PEO CNT/PCL). The experimental results show that the surface produced by PCL deposition is the smoothest and reduces magnesium corrosion. The effects of the above three materials/surfaces on the metabolism of bone marrow mesenchymal stem cells (BMSCs) were investigated, and it was found that the cells of the PEO CNT/PCL test group formed very fine mesh flake pseudopodia with a wide and thin adhesion surface, and the surface was smooth and uniform, which was conducive to cell attachment, proliferation, and osteogenic differentiation [80]. Duong coated the polymer layer of gelatin/nano-hydroxyapatite (Gel/nHA) on the orthopedic implant ZK60 magnesium (Mg) alloy through dipping and spin coating, and found that the cell proliferation and corrosion resistance were enhanced, and the cells had better hemolysis and antiplatelet adhesion [81]. Degradable coatings have proven to be a viable and adaptable option for bioactive metals. Due to their high biocompatibility and biodegradability, they are considered a promising scaffold material with a low risk of long-term side effects, which can be gradually degraded and used in regenerative medicine and tissue engineering.

#### 2.1.5 Coating fabrication and surface modification technologies

The performance of bioactive metals, specifically the bonding strength, layer uniformity, and bioactivity release is critically dependent on the fabrication and modification technologies employed [82]. These technologies can be broadly categorized into coating deposition methods and electrochemical surface modifications (Table 1).

**Table 1:** Coating deposition methods and surface modification technologies.

Classification	Materials	Advantages	Ref.
Thermal spraying	Hydroxyapatite coating	Enhance biocompatibility and osteogenic differentiation of cells	[35]
Sol-gel method	Cu-BG coating	Thick or thin coatings can be prepared to achieve gradient controlled release of functional ions	[45,47]
Magnetron sputtering	AgNP coatings	Achieve the uniform distribution of antibacterial nanoparticles on the surface	[59]
Dipping technology	CS/Gel/AHA hydrogel coating	For local drug delivery and anti-biofilm	[77]
Spin-coating	PCL coating (Magnesium alloy)	Provide effective corrosion protection and significantly reduce the corrosion rate.	[79]
Anodic oxidation	TiNbSn	Give the material antibacterial properties	[83]
	Ti6Al4V	Suppress the release of metal ions (Ti/Al/V) and enhance the corrosion resistance of the material	[84]
	TiO_2_	Promote osteogenic differentiation	[85]
Plasma electrolytic oxidation	Magnesium alloy X0	Inhibit gas escape after implantation and promote bone integration	[86]
	SrO/SrTiO_3_/Sr_3_(PO_4_)_2_ coatings	Promote bone formation	[87]
Electrochemical deposition	HAp-Nb_2_O_5_ composite coating	Enhance the corrosion resistance and antibacterial properties by inducing apatite formation and inhibit bacterial adhesion	[88]
	A polypyrrole base	Promote the early adhesion of osteoblasts	[89]
	Ferroelectric KNNCu	Increase antibacterial efficiency from 65% to 100% and maintain low cytotoxicity	[90]

Different strategies are selected based on the coating material and substrate geometry. Thermal spraying utilizes heat sources (flame, plasma) to melt and propel coating materials onto the substrate, a method widely used for creating thick, robust HA coatings [35]. For precise, nanoscale thin films, magnetron sputtering is employed; this technique uses magnetic fields and high-energy ions to bombard a target, depositing dense and highly adhesive metallic or oxide films (e.g., AgNPs) [59]. The sol-gel method offers a chemical route, involving the hydrolysis and polycondensation of precursors to form a gel network, which is ideal for synthesizing high-purity bioactive glass or ceramic coatings with molecular homogeneity [45]. For polymer and composite layers, solution-based techniques are preferred: dipping technology involves immersing and withdrawing the substrate to form coatings on complex geometries through viscosity and surface tension [77], while spin-coating utilizes centrifugal force to spread solutions on flat surfaces, achieving highly uniform thickness control [79].

In addition to deposition, surface modification technologies are crucial for enhancing the interface between the coating and the metal matrix: anodic oxidation can build an oxide layer on the metal surface through electrolyzing an oxygen-containing solution [91], which can promote bone formation and differentiation, and make the metal implant have a broad spectrum of antibacterial activity and good corrosion resistance. PEO forms a composite ceramic layer to promote bone integration through high-voltage micro-arc discharge. Electrochemical deposition can be used to achieve the precise deposition of functional coatings to enhance the corrosion resistance and antibacterial properties of metal implants, and promote the early adhesion of osteoblasts. Different surface modification techniques can improve the different properties of metal implants and promote the long-term stability of implants.

Bone implant coatings still face numerous challenges including long-term stability in body fluids, precise and sustained drug release, and multifunctional synergistic design (antibacterial/anti-inflammatory/bone-forming), along with clinical translation difficulties such as degradation matching, immune adaptation, and personalized customization [92,93]. Currently, antimicrobial coatings on biomedical implant surfaces are evolving towards intelligent response and multifunctional synergy. Intelligent release systems enable precise regulation through environmental triggers, thereby enhancing implant corrosion resistance, antibacterial and anti-inflammatory capabilities, significantly promoting osteoblast proliferation and differentiation, improving biocompatibility, and precisely treating infectious bone defects [[94], [95], [96], [97], [98], [99], [100], [101]]. Additionally, innovations in coating preparation technologies-such as ML, electrophoretic deposition techniques, and the construction of micro-nano hierarchical structures-are effectively supporting the integration of multifunctional capabilities [[102], [103], [104], [105], [106], [107], [108], [109]].

In summary, implants hold significant importance in the biomedical field, with various bioactive coatings demonstrating tremendous potential in tissue engineering applications. Through multiple surface modification techniques, these coatings not only overcome the limitations of metallic implant materials but also fully leverage their advantages. However, bioactive coatings still face challenges such as high infection risks, insufficient bonding strength, and poor biocompatibility, requiring cross-disciplinary collaborative research. In the future, intelligent response mechanisms, multifunctional synergistic effects, and nanotechnology will become crucial development directions. Only through in-depth exploration of these technologies can we fully realize their medical value, thereby enhancing the long-term stability and clinical efficacy of implants.

### 2.2 Bioactive ceramics: bioglass, calcium phosphate

Bioactive ceramics represent a class of functional materials capable of forming chemical bonds with host tissues in physiological environments [110,111]. Their defining characteristic lies in actively regulating the bone regeneration microenvironment through controlled ion release and surface reactions [112]. Since Larry Hench's invention of the first BG (45S5 Bioglass®) in 1969, these materials have progressively replaced traditional bioinert implants to become the mainstream choice in bone defect repair [113]. Unlike conventional metals or polymers, bioactive ceramics exhibit three distinct biological properties: osteoconductivity (providing a physical scaffold for bone cell adhesion and growth), osteoinductivity (activating osteogenic differentiation pathways via ionic signaling), and surface biomineralization capacity (forming a bone-like apatite layer at the interface) [114]. Their bioactive mechanism originates from a dynamic dissolution-reprecipitation process in bodily fluids: ions such as calcium, phosphorus, and silicon released from the material surface participate in local biochemical reactions, inducing the deposition of a HA crystalline layer [115]. This layer shares compositional similarity with natural bone mineral phases, serving as a bridge for tissue integration. Current bioactive ceramic systems primarily comprise silica-based bioglasses and calcium phosphate ceramics-the former achieve high reactivity through amorphous structures, while the latter mimic bone mineral composition via crystalline phase design [116].

**Fig. 4:**
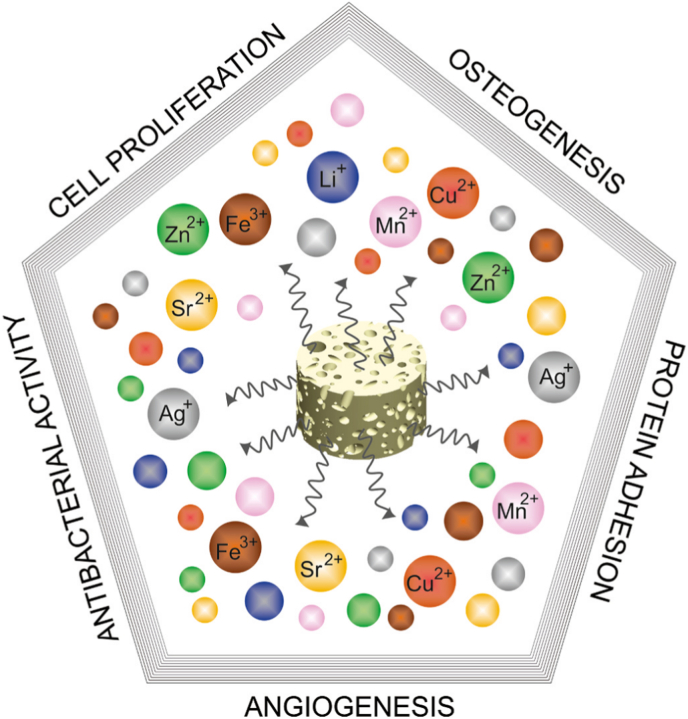
Schematic representation of biological responses to metal ions on bioceramics [125]. Various metal ions (e.g., Li^+^, Zn^2+^, Sr^2+^, Ag^+^, Cu^2+^, Mg^2+^, Fe^3+^, Mn^2+^) released from bioceramic scaffolds trigger key biological responses, including osteogenesis, cell proliferation, antibacterial activity, angiogenesis, and protein adhesion, which are critical for bone tissue regeneration and implant integration.

BG, also known as bioglass, refers to a category of surface-reactive inorganic non-metallic biomaterials capable of human tissue repair, functional replacement, and regeneration, facilitating bonding between tissues and the material. The classic 45S5 formulation (45% SiO_2_, 24.5% CaO, 24.5% Na_2_O, 6% P_2_O_5_) undergoes the following osseointegration reactions upon implantation: When exposed to physiological fluids, alkali metal ions (e.g., Na^+^) at the material surface rapidly exchange with hydrogen ions (H^+^ or H_3_O^+^), forming a silica-rich gel layer [117]. Subsequently, calcium and phosphate ions deposit and nucleate on this layer, crystallizing into HA crystals. Ultimately, this biomineralized layer crosslinks with bone collagen fibers, establishing a bonded interface with strength exceeding that of natural bone. Calcium phosphate ceramics represent a class of crystalline biomaterials primarily composed of HA (Ca_10_(PO_4_)_6_(OH)_2_) and TCP (Ca_3_(PO_4_)_2_). Their calcium-to-phosphorus ratio (1.5-1.67) closely matches the mineral composition of human bone, enabling bioactivity through a dissolution-reprecipitation mechanism in physiological environments [118,119]. Upon implantation, these materials progressively release calcium and phosphate ions from their surfaces. Under localized supersaturation conditions, this ionic release induces the deposition of HA. The resulting biomineralized layer forms chemical bonds with host bone collagen while simultaneously providing an osteoconductive scaffold. Despite significant advancements in bioceramic materials, precise regulation of their biological functions still faces substantial challenges: the burst release of ions from bioceramics may trigger localized pH fluctuations; a mismatch exists between the degradation rate of calcium phosphate ceramics and bone regeneration kinetics [120]; conventional bioceramics lack disease-specific functionalities (e.g., antibacterial activity, immunomodulation) [121,122]; clinical treatments demand multifunctional bioceramics (photothermal, piezoelectric, and magnetothermal effects) [117,123,124]. These limitations have driven researchers to shift their focus toward metal ion doping engineering-by introducing specifically valenced metal ions to modify the bioceramics, achieving a paradigm shift from “single-function bioceramics” to multifunctional bioceramic platforms (Fig. 4) [125].

#### 2.2.1 Strontium ions

Strontium (Sr) is recognized as a naturally bone-seeking element due to its physical and chemical similarities to calcium, enabling it to substitute calcium as a bone-mimetic matrix. Given the excellent osteogenic and angiogenic properties of strontium ions (Sr^2+^), they have been extensively utilized as dopants in bioceramics. Zhuang and co-workers successfully synthesized multifunctional strontium-incorporated hydroxyapatite (Sr-HA) bioceramics via chemical precipitation. They verified that Sr^2+^ not only enhances osteogenic differentiation of BMSCs through Erk1/2 and PI3K/AKT signaling pathways but also activates the PI3K/AKT pathway in human umbilical vein endothelial cells (HUVECs) to promote angiogenesis [126]. Another study by Zhao and co-workers found that strontium-containing bioactive glasses microspheres (SrBGM) can regulate macrophage polarization to promote vascularization [127]. The ions released from SrBGM significantly increase the tendency of macrophage polarization from M1 to M2 phenotype and promote platelet-derived growth factor-BB (PDGF-BB) secretion by macrophages, thus facilitating blood vessel formation and maturation. This finding elucidates the cascading mechanism of “immunomodulation-angiogenesis-osteogenesis” mediated by strontium ions, offering a novel therapeutic strategy for complex bone defect repair. Additionally, Sr^2+^ competitively inhibits the receptor activator of nuclear factor κB ligand (RANKL) pathway to suppress osteoclast activity, thereby ameliorating osteoporosis. For example, the strontium-doped calcium phosphate ceramics (SrCaP) developed by Lu and co-workers not only significantly promote the osteogenic differentiation of stem cells but also reduce tartrate-resistant acid phosphatase (TRAP) enzymatic activity in osteoclasts [128]. Among them, the SrCaP scaffold with 30 mol.% strontium doping (Sr30-S) demonstrated the best performance in promoting osteogenic differentiation and inhibiting osteoclast formation. In osteoporotic bone defects repairing, strontium-doped bioceramics overcome traditional limitations through integrated “composition-structure-function” design. Zhao and co-workers successfully prepared strontium-doped hydroxyapatite whisker bioceramics (SrWCP), which can release Sr^2+^ under physiological conditions to mitigate osteoporosis-induced bone loss [129]. Furthermore, Song and co-workers combined icariin with strontium-doped bioceramic scaffolds to create a self-crosslinking functionalized system [130]. In osteoporotic rat models, this scaffold exhibited multiple synergy: Sr^2+^ directly stimulated osteogenic gene expression, icariin activated the BMP-2/Smad pathway, and silicon ions (Si^4+^) promoted vascular endothelial growth factor (VEGF) secretion.

#### 2.2.2 Magnesium ions

Magnesium (Mg) is an essential mineral element in bone metabolism, playing a critical role in bone mineralization and regulating the activity of both osteoblasts and osteoclasts [131]. Consequently, magnesium ions (Mg^2+^) have also been incorporated into various bioceramics as dopants to enhance their biological functionality, and the efficacy of magnesium-containing bioceramics in bone repair has been well-documented [132]. For example, Ballouze and co-workers introduced Mg^2+^ into biphasic calcium phosphate (BCP), which not only markedly improved the mechanical properties of bioceramics, but also endowed the Mg-BCP scaffold with excellent bone-regenerative capacity in vivo [133]. Beyond excellent biocompatibility, Mg^2+^ also possesses strong pro-angiogenic potential. Ho and co-workers fabricated a 3D-printed magnesium/strontium co-doped calcium silicate scaffolds. The released Mg^2+^ from scaffolds fosters early-stage neovascularization, thereby laying the groundwork for subsequent Sr^2+^-enhanced osteogenic differentiation [134]. Moreover, studies have revealed that Mg^2+^ can modulate immunity by inhibiting osteoclastogenesis. Bose and co-workers seeded osteoclast precursor cells (RAW264.7) onto Mg^2+^ doped β-TCP substrates and found that the cells failed to form actin rings, while the expression of osteoclastic marker genes such as TRAP and nuclear factor of activated T-cells cytoplasmic 1 (NFATc1) was markedly down-regulated [135]. Mg^2+^ also exhibits antibacterial activity. The MgO-HA bone substitute developed by Coelho and co-workers creates a localized alkaline microenvironment in physiological fluid while releasing reactive oxygen species (ROS)-scavenging electrons, resulting in significant inhibition of both *Staphylococcus aureus* and *Escherichia coli* and thereby lowering the risk of early-stage infection. Consequently, Mg^2+^ in bone-regeneration applications exhibits an optimized combination of antibacterial and pro-angiogenic functions [136].

#### 2.2.3 Iron ions

Iron ions (Fe^3+^/Fe^2+^) are essential trace elements in the human body and exhibit excellent biocompatibility. Bioceramics incorporating iron ions not only preserve the good biocompatibility of the bioceramics but also enhance their capacity to promote tissue regeneration and repair. For example, researchers utilized calcium ammonium ferric citrate to modify calcium phosphate cement. The introduced iron ions not only regulate the hydration process of the cement to enhance its compressive strength but also impart favorable osteogenic efficacy in vivo [137,138]. Furthermore, the doping of iron ions imparts magnetic and photothermal properties to the bioceramics. When combined with external magnetic fields and near-infrared irradiation, bioceramics significantly enhanced osteogenic differentiation of stem cells, modulated macrophage polarization, and synergistically promoted angiogenesis, thereby offering a drug-free, remotely controllable strategy for bone-defect repair. For instance, the 3D-printed ferric gallate (FeGA)-modified HA nanowire scaffold developed by Yin and co-workers combines both photothermal antibacterial and chemodynamic effects under near-infrared irradiation [139]. This scaffold eliminates infection while enabling sustained release of low-dose Fe^3+^ ions, thereby reversing the iron-deficient microenvironment and inducing macrophage polarization toward the M2 phenotype. Zhang and co-workers synthesized superparamagnetic iron-doped calcium phosphate cement (Fe-CPC) that exhibits excellent biocompatibility. BMSCs cultured on the Fe-CPC displayed favorable adhesion, robust proliferation, and enhanced osteogenic differentiation. When remotely stimulated with a 100 mT static magnetic field, the Fe-CPC markedly increased the bone mineral density of mouse calvarial defects [140]. In addition, iron-doped bioceramics also play a pivotal role in the field of tumor therapy [141]. Iron-doped bioceramics achieve synergistic antitumor efficacy primarily through iron-mediated magnetothermal therapy, photothermal therapy, and induction of ferroptosis [[142], [143], [144], [145]].

#### 2.2.4 Other metal ions

In addition, metal ions such as zinc, copper, silver, cobalt, and lithium have also been used to enhance the bioactivity of bioceramics. Their incorporation endows bioceramics with a rich array of functions, such as promoting vascularization, modulating immunity, enhancing neurogenesis, exerting antibacterial effects, and suppressing tumors. For instance, Cu^2+^ and cobalt ions (Co^2+^) are pivotal regulators of vascularization. Co^2+^-doped calcium phosphate directly stimulates endothelial cell migration by activating the hypoxia-inducible factor 1 alpha (HIF-1α)/VEGF pathway, whereas Cu^2+^-doped calcium silicate markedly increases neovascular density by stabilizing HIF-1α through mimicking a hypoxic environment [146,147]. The incorporation of Ag^+^ and Cu^2+^ endows bioceramics with excellent antibacterial properties. Ag^+^-doped HA achieves a bacteriostatic rate exceeding 99 % against methicillin-resistant *Staphylococcus aureus* (MRSA), while Cu^2+^ embedded in zirconia ceramics disrupts bacterial biofilms; both effectively prevent post-implantation infections [148,149]. Doping with Mn^2+^ and Co^2+^ further enhances the antitumor efficacy of bioceramics. Mn^2+^-doped bioceramics raise the local temperature to 50 °C via photothermal conversion, whereas Co^2+^ disrupts metal homeostasis and synergizes with ferroptosis [150,151]. In addition, lithium (Li^+^) and zinc (Zn^2+^) ion-doped bioceramics play a pivotal role in neural regeneration [152]. Wei and co-workers found that extracts from Li^+^-doped BG promoted Schwann cell proliferation and migration [153].

### 2.3 Bioactive polymers: natural polymers and synthetic polymers with bioactive moieties

Bioactive polymers are a class of functional macromolecular materials capable of specific interactions with living systems to induce or regulate particular biological responses. These materials not only retain the structural characteristics of conventional polymers but can also achieve active interfacing with biological tissues through surface chemical modification, functional group incorporation, or specific structural designs [154]. Their bioactivity is primarily manifested in promoting cell adhesion, proliferation and differentiation, inducing tissue regeneration, or producing therapeutic effects such as antimicrobial and anti-inflammatory actions. Based on molecular mechanisms, bioactive polymers can be classified into direct-active types (containing specific biological signal molecules) and indirect-active types (influencing cellular behavior through surface topographical features). From the perspective of origin, they are divided into two major categories: natural bioactive polymers including collagen, hyaluronic acid, and fibrinogen derived from organisms, which exhibit inherent biomolecular recognition and excellent tissue compatibility [[155], [156], [157]]; and synthetic bioactive polymers such as polyethylene glycol (PEG) derivatives, polyamino acids, and phosphorylcholine-based polymers prepared through chemical synthesis, featuring precisely tunable molecular structures and good batch-to-batch consistency [158,159]. Through molecular design or composite modification, these two categories of materials play indispensable roles in biomedical applications including drug delivery systems, tissue engineering scaffolds, and medical implants [160]. Their bioactive performance depends not only on the material's chemical composition but also on physical characteristics such as microscopic morphology and surface energy.

Natural bioactive polymers (such as hyaluronic acid, collagen, and fibrinogen) are demonstrating vast potential in tissue engineering and regenerative medicine due to their outstanding biocompatibility, biodegradability, and biomimetic properties. Beyond faithfully recapitulating the extracellular matrix (ECM) microenvironment to facilitate cell adhesion, proliferation, and differentiation, these materials leverage their intrinsic functional groups to engage in biological signal transduction, thereby orchestrating the entire tissue-regeneration process. For instance, hyaluronic acid is a primary constituent of cartilage. Researchers have employed genetic engineering to fabricate recombinant collagen scaffolds with precisely graded mechanical properties that faithfully replicate the osteochondral interface [161]. In osteoarthritis repair, these scaffolds have achieved simultaneous regeneration of both cartilage and subchondral bone. Keilhoff and co-workers employed type I/III collagen tubes as templates for “living” nerve conduits. By seeding Schwann cells into their lumens, they successfully bridged extended nerve gaps and ensured nerve regeneration [162]. Moreover, researchers have further enhanced the mechanical performance and biological functions of these materials through molecular modification, composite cross-linking, and micro-/nano-structural design. Gallagher and co-workers pre-cultured MSCs in Arg-Gly-Asp (RGD)-functionalized HA hydrogels, significantly enhancing cell survival and functional recovery in ischemic lesions. Compared with controls, the HA-RGD scaffold markedly promoted MSC spreading and augmented the release of VEGF-1 and monocyte chemoattractant protein-1 (MCP-1) [163]. As the deacetylated derivative of chitin, CS serves as an ideal bioactive carrier owing to its cationic nature and pH-responsive solubility. In an infected microenvironment (pH < 6), CS rapidly releases drugs and disrupts bacterial membranes, yielding >95 % inhibition against *Escherichia coli.* Concurrently, ALP-triggered dephosphorylation liberates calcium and phosphate ions locally to promote mineralization [164]. Furthermore, Li and co-workers fabricated an interpenetrating polymer network scaffold composed of collagen, hyaluronic acid, and chondroitin sulfate for brain tissue engineering [165]. Compared with single-polymer scaffolds, the composite material exhibited markedly enhanced neurogenesis, offering significant advantages for cerebral repair in tissue-engineering therapies. These innovative studies not only confirm the pivotal role of natural bioactive polymers in tissue repair, but also provide crucial insights for the development of next-generation smart biomaterials.

Synthetic bioactive polymers enable precise functional customization through programmable molecular architectures, offering distinct advantages in mechanical properties, degradation kinetics, and bioactivity regulation. PEG, the “gold-standard” biomaterial, is widely used for surface coatings because of its hydrophilicity and non-fouling properties. Terminal functionalization with -COOH or -NH_2_ allows covalent conjugation of the cRGDfk cyclic peptide, enabling specific binding to active tumor neovasculature for targeted tumor ablation [166]. Nevertheless, the non-degradable nature of PEG constrains its long-term applications. Poly (lactic-co-glycolic acid) (PLGA) enables precise control of degradation time by tuning the LA/GA ratio. Its hydrophobic backbone encapsulates runt-related transcription factor 2 (Runx2) protein via an emulsion method, while the surface is engineered to present BMP-2-encoding plasmids, creating a temporally controlled release system that effectively drives human mesenchymal stem cells (hMSCs) toward osteoblast differentiation [167]. Moreover, synthetic bioactive polymers have been engineered into stimuli-responsive smart materials. For example, poly (N-isopropylacrylamide) (PNIPAM) undergoes a sol-gel transition at 32 °C. Copolymerization with N-hydroxyethyl acrylamide precisely raises its lower critical solution temperature (LCST) to 37 °C, enabling the encapsulation of MSCs for in situ filling of bone defects [168]. Driven by advances in AI-assisted molecular design (such as predicting polymer-protein binding energies) and 4D printing (with thermo- or photo-actuated shape transformation), synthetic bioactive polymers are accelerating toward truly personalized precision medicine [[169], [170], [171]].

### 2.4 Bioactive composites

**Fig. 5:**
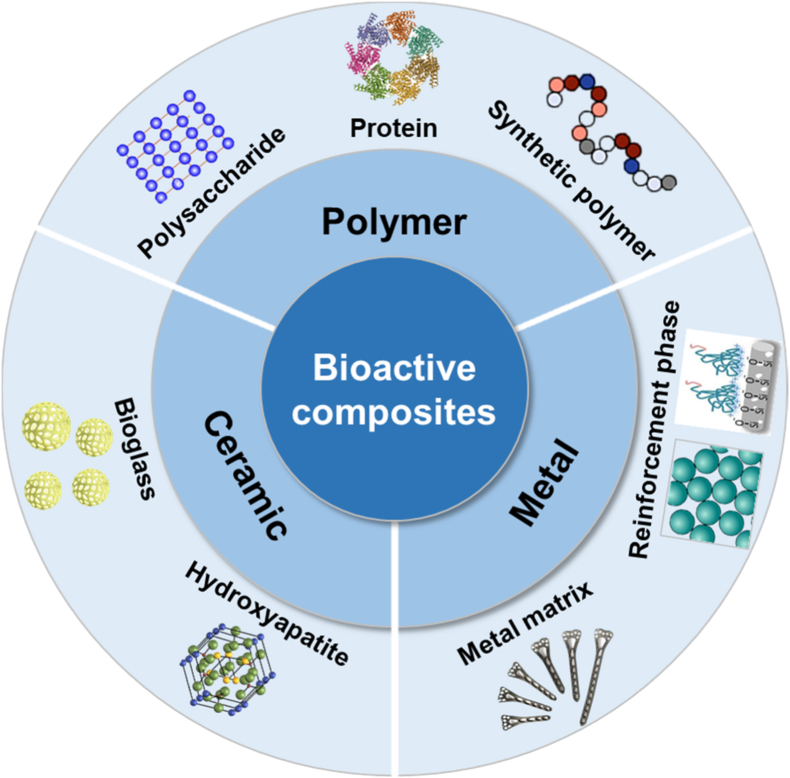
Classification of bioactive composites of polymer, ceramic and metal. The polymer includes polysaccharides, proteins, and synthetic polymers. The ceramic encompasses hydroxyapatite and bioglass. The metal features metal matrices and reinforcement phases.

Bioactive composites are a class of advanced biomaterials capable of undergoing specific reactions with biological tissues (particularly bone tissue), forming chemical bonds, while possessing tunable mechanical properties and degradation characteristics. Their classification is diverse and primarily based on the type of matrix material, categorized into polymer-based, ceramic-based, and metal-based composites (Fig. 5).

#### 2.4.1 Polymer-based composites

The high hydrophilicity, biodegradability, and similarity to the ECM of polysaccharide-based natural polymers have garnered significant attention in the biomedical field. Firstly, high hydrophilicity allows these polysaccharides to form hydrogen bonds with water molecules, enhancing their hydration and enabling the formation of stable colloids or hydrogels in water [172]. At the cellular microenvironment level, this highly hydrated network facilitates nutrient diffusion, oxygen transport, and metabolic waste removal, thereby supporting cell survival and functional activity within three-dimensional scaffolds [173]. For instance, hyaluronic acid, due to the carboxyl and hydroxyl groups in its structure, effectively absorbs water and forms biocompatible hydrogels. This property is crucial for the development of tissue engineering scaffolds and drug delivery systems. Secondly, the biodegradability of natural polysaccharides allows them to be enzymatically degraded within the body, avoiding the issues of bioaccumulation and potential toxicity associated with synthetic polymers. This characteristic makes polysaccharide-based polymers ideal choices for biomedical materials, particularly in drug delivery and tissue regeneration, where they can effectively release bioactive substances without causing toxic side effects [174]. Importantly, the degradation process can be coupled with controlled release of encapsulated drugs or signaling molecules, enabling spatiotemporally regulated therapeutic effects that better match tissue regeneration kinetics [175]. Finally, the similarity of natural polysaccharides to the ECM enables them to mimic the in vivo environment, promoting cell adhesion and proliferation. For instance, CS's structure resembles glycosaminoglycans in the ECM, allowing it to effectively facilitate interactions between cells and the material, thereby enhancing tissue regeneration outcomes [176]. This similarity endows polysaccharide materials with broad application prospects in regenerative medicine, tissue engineering, and drug delivery.

Protein-based natural polymers promote cell adhesion, proliferation, and differentiation through multiple mechanisms. Firstly, the surface chemistry and three-dimensional structure of collagen and silk fibroin provide favorable attachment sites for cells, promoting adhesion to their surfaces. Studies show that collagen can bind to cell surface receptors like integrins, activating downstream signaling pathways that promote cell proliferation and migration [177]. This integrin-mediated interaction triggers intracellular pathways such as focal adhesion kinase (FAK) and MAPK signaling, which are critical for cytoskeletal reorganization and lineage-specific differentiation [178]. Furthermore, degradation products of collagen (such as small peptides) can also stimulate cell proliferation and migration, creating a favorable biological environment for tissue regeneration.

The biodegradability of protein-based natural polymers is a key characteristic for their use in tissue engineering applications. Collagen and silk fibroin degrade within the body via enzymatic and chemical pathways. Notably, the degradation rate of protein-based scaffolds can be modulated by molecular crosslinking or secondary-structure regulation, allowing mechanical support to persist until newly formed tissue achieves sufficient strength [179].

Enzymatic degradation primarily involves specific enzymes (like collagenase and fibroinase) cleaving their peptide chains into small peptides or amino acids. These degradation products can be absorbed by cells and utilized in the synthesis of new tissues [180]. Additionally, the degradation rate of proteins can be modulated through molecular design and modification to suit different tissue regeneration requirements. In tissue engineering, protein-based polymers are used as scaffold materials, providing a matrix for cell attachment and proliferation. For example, collagen scaffolds can promote the regeneration of cartilage and bone tissue and can be combined with growth factors to enhance their bioactivity [177]. Silk fibroin, owing to its superior mechanical properties and excellent biocompatibility, is widely applied in the repair and regeneration of various tissues, including nerves, bone, and muscle. Its scaffolds can be fabricated using 3D printing technology to meet complex shape and functional needs [180].

The application of synthetic polymers in the biomedical field is increasingly widespread [[181], [182], [183]]. PLA, PCL, polyvinyl alcohol (PVA), and PEG are several commonly used synthetic polymers. Regarding physical properties, PLA and PCL have relatively high melting points and good mechanical properties, making them suitable for load-bearing biomaterials. In contrast, PVA and PEG exhibit better water solubility and biocompatibility, making them suitable for drug delivery and the preparation of biological membranes. The biodegradability of PLA gives it broad application prospects in the biomedical field, particularly in the manufacture of absorbable sutures and drug release systems, where it demonstrates significant value [184]. PCL also shows good adaptability in tissue engineering due to its tunable degradation rate, which can be adjusted to suit different biological environments [185].

#### 2.4.2 Ceramic-based composites

Over the past four decades, BGs have evolved into a cornerstone biomaterial platform for osseous regeneration, as exemplified by the clinically established melt-derived 45S5 composition [[186], [187], [188]] and the chemically versatile sol-gel-derived 58S system [189,190]. Emerging evidence has demonstrated the enhanced therapeutic efficacy of drug delivery systems based on MBGs. Prior research confirms that BG ionic dissolution products stimulate osteogenic differentiation and enhance bone regeneration [188]. These released ions act as biological cues that upregulate osteogenic gene expression (e.g., Runx2, ALP, and OCN) and promote angiogenesis, thereby coupling bone formation with vascularization [191]. Recent studies demonstrate that therapeutic-ion-doped MBGs can promote osteoblast differentiation. For instance, Cu-MBGNs could promote bone defect healing by enhancing osteoblast autophagy and mitophagy through ROS-mediated mitochondrial fission, thereby accelerating amorphous calcium phosphate mineralization [192]. Another study demonstrates that Ce-MBGNs scavenge excess ROS and restore oxidative homeostasis via Kelch-like ECH-associated protein 1 (Keap1)/Nuclear factor erythroid 2-related factor 2 (Nrf2) pathway modulation, thereby promoting osteogenic differentiation under diabetic conditions [193]. These findings highlight that ion-doped MBGs exert osteogenic effects not only through structural support but also by actively regulating intracellular redox signaling pathways [132]. In addition, MBGNs can be loaded with therapeutic agents such as gentamicin, ampicillin, and ibuprofen to achieve targeted antibacterial effects through distinct mechanisms [188,194].

Despite compelling osteogenic potential, BG confronts persistent translational barriers. There are two fundamental knowledge gaps that impede progress. One is the unvalidated co-delivery synergism: whether ionic/pharmaceutical co-release from MBGs synergistically enhances in vitro osteogenic differentiation and in vivo critical-sized defect regeneration awaits systematic validation. The other is the undefined cooperative mechanisms: how therapeutic ions and pharmaceuticals collectively drive multifunctional properties remains mechanistically unresolved.

HA exhibits exceptional biocompatibility and bioactivity, attributable to its compositional and structural similarity to the inorganic component of biological apatite in natural bone. Ionic doping of HA, particularly with Sr^2+^ and Mg^2+^, enhances its bone regenerative efficacy by strategically modifying crystal structure and bioactivity. Sr^2+^ promotes osteoblast proliferation and differentiation while simultaneously suppressing osteoclast activity, thus modulating bone remodeling toward net formation. Mg^2+^ acts as an essential cofactor in calcium metabolism and directly stimulates bone mineralization. Consistent with these mechanisms, strontium-doped HA demonstrates superior osteogenic performance both in vitro and in critical-sized bone defect models in vivo. Consequently, targeted ionic substitution represents a strategic approach to optimize HA's bioactivity for advanced bone regeneration applications [195,196].

The formation of HA composites with polymers such as PLA or collagen substantially enhances the material's mechanical properties. These composites effectively retain HA's essential bioactivity while simultaneously mitigating its inherent brittleness and improving fracture toughness and strength, enabling reliable performance under physiological loading conditions. For instance, PLA-a biocompatible synthetic polymer-significantly increases the compressive and tensile strength of HA composites while maintaining excellent cytocompatibility. Multiple studies confirm that the mechanical properties of optimized HA/polymer composites align closely with those of natural cancellous bone, establishing a solid biomechanical foundation for bone repair, particularly in load-bearing applications. Critically, the mechanical behavior and degradation profile of these composites are precisely tunable through systematic adjustments to the polymer type, content ratio, molecular weight, and crystallinity [197,198].

#### 2.4.3 Metal-based composites

Metal matrix composites (MMCs) are composite materials composed of a metallic matrix and a reinforcement phase, exhibiting exceptional mechanical properties and corrosion resistance. The metallic matrix typically provides structural strength and toughness to the material, while the reinforcement phase enhances overall performance by improving strength, hardness, and wear resistance. At the microstructural level, reinforcement phases impede dislocation movement and redistribute mechanical stress, thereby significantly improving fatigue resistance and long-term structural stability [199]. Reinforcement phases may take the form of particles, fibers, or other geometries-commonly including ceramic particles, metallic fibers, or carbon nanotubes. By integrating the distinct properties of different constituents, this composite system enables targeted optimization of material properties for specific application requirements [200]. Magnesium alloys are considered excellent candidates for biodegradable metal implants, drawing significant attention due to their superior mechanical properties and biocompatibility. However, as hydrogen evolution during the degradation process may interfere with tissue regeneration, surface modification becomes essential to optimize their stability and bioactivity [201]. Through techniques such as plasma PEO, dense ceramic coatings can be formed on magnesium alloy substrates, significantly enhancing their biocompatibility and mechanical properties [202]. These coatings act as diffusion barriers that slow corrosion kinetics while simultaneously providing bioactive surfaces that promote osteointegration [203].

In the biomedical field, MMCs attract particular attention due to their capability to deliver exceptional mechanical properties and biocompatibility. Titanium alloys and stainless steel represent two primary metallic substrates. While titanium alloys are frequently utilized in bone implants owing to their low density and high biocompatibility, stainless steel is predominantly used in medical devices and surgical implants because of its superior corrosion resistance and mechanical strength. The high strength coupled with excellent oxidation resistance of titanium alloys renders them ideal for biomedical applications, especially when substantial mechanical loads must be withstood [204]. At the biological interface, titanium alloys spontaneously form a stable TiO_2_ passive layer, which not only confers corrosion resistance but also modulates protein adsorption and osteoblast adhesion, thereby promoting osseointegration at the bone–implant interface [205]. Stainless steel is extensively employed in surgical instruments and implants owing to its cost-effectiveness and manufacturability, enabling it to cater to diverse biomedical applications [206].

The bonding strength between the coating and the metal substrate is a crucial factor affecting the performance of composite materials, especially in biomedical applications. The main reasons for insufficient adhesion of the coating include poor chemical compatibility between the coating material and the substrate material, improper surface treatment, and inappropriate coating application methods [207]. For example, PVA coatings exhibit poor adhesion due to inadequate interfacial interaction with the metal substrate. To improve this situation, surface modification techniques can be employed, such as applying a metal plating or using chemical treatments to enhance the bonding strength between the coating and the substrate [208]. Additionally, incorporating nanofillers such as carbon nanotubes and graphene can enhance the coating's adhesion and wear resistance to some extent, thereby improving the overall mechanical properties and durability of the composite material. From a mechanistic perspective, nanofillers increase interfacial load transfer efficiency and inhibit crack initiation and propagation through nanoscale reinforcement and interlocking effects, which is particularly critical under cyclic physiological loading conditions [209].

In the biomedical domain, the wear resistance and corrosion resistance of metal matrix composite coatings serve as critical metrics for evaluating their performance in physiological environments. The durability of these coatings directly dictates the service life and biocompatibility of implant materials, consequently impacting patient recovery outcomes. Research demonstrates that TiO_2_ coatings formed via PEO on titanium alloy (Ti20Nb20Zr4Ta) substrates exhibit remarkably superior tribological and anti-corrosion properties. These coatings not only enhance the alloy's biocompatibility but also possess inherent antimicrobial characteristics that reduce infection risks in vivo [210].

Furthermore, a coating's resorption kinetics must be temporally coordinated with the regeneration rate of surrounding tissues to ensure persistent therapeutic functionality throughout the implant's in vivo lifespan. This synchronization prevents premature structural collapse before tissue remodeling completes or prolonged foreign body effects post healing [211].

### 2.5 Summary of bioactive materials

In summary, this section provides a comprehensive overview of major bioactive materials, including metals with bioactive coatings, ceramics, polymers, and composites. These materials can actively modulate cell behavior and immune responses, facilitating tissue regeneration through controlled ion release, surface activity, and structural design. Although significant advances have been achieved, challenges remain in controlling degradation, ensuring long-term stability in vivo, and integrating multiple functions without compromising biocompatibility. Addressing these issues increasingly requires the application of AI to guide the rational design and optimization of bioactive materials.

## 3 AI tools in bioactive materials

The aim of this section is to introduce the field of AI as well as to highlight the potential of AI methods within materials science and engineering with an emphasis on the development and investigation of bioactive materials. One of the accepted definitions of the term *artificial intelligence* can serve as a starting point for further consideration. This definition of AI states that: “AI is the field of computer science that creates systems able to learn, reason, and act intelligently”. Independently from the field of activity, AI algorithms are usually applied to phenomenon **classification** and **regression**. The former is mainly used for assigning an input to one of several predefined classes, such as image recognition, materials features detection, etc. The latter aims to predict a numerical quantity, such as material strength or material degradation progression. AI algorithms may also serve as advisory systems, a role that does not exclude any of the previously mentioned functions. The presented set of functions is not exhaustive and remains somewhat arbitrary, but it highlights the most frequently used applications. Some algorithms can perform several of these functions effectively, while others demonstrate strong performance only in specific tasks.

This section is organized as follows. It begins with a presentation and explanation of the most fundamental taxonomies used to categorize AI methods, namely hierarchical taxonomy and taxonomy based on learning approach. These provide the conceptual foundation for a detailed introduction to the most widely used machine learning methods in bioactive materials engineering and research. All discussed methods are further classified according to a functional taxonomy specific to the bioactive materials field. AI-based QSAR tools are described in a separate subsection, given their particular importance in the design and development of new materials and drugs.

### 3.1 General classification of AI methods

Several taxonomies can be used to categorize AI methods. Below, two of the most fundamental are presented: the hierarchical taxonomy and the taxonomy based on the learning approach.

#### 3.1.1 Hierarchical taxonomy

**Fig. 6:**
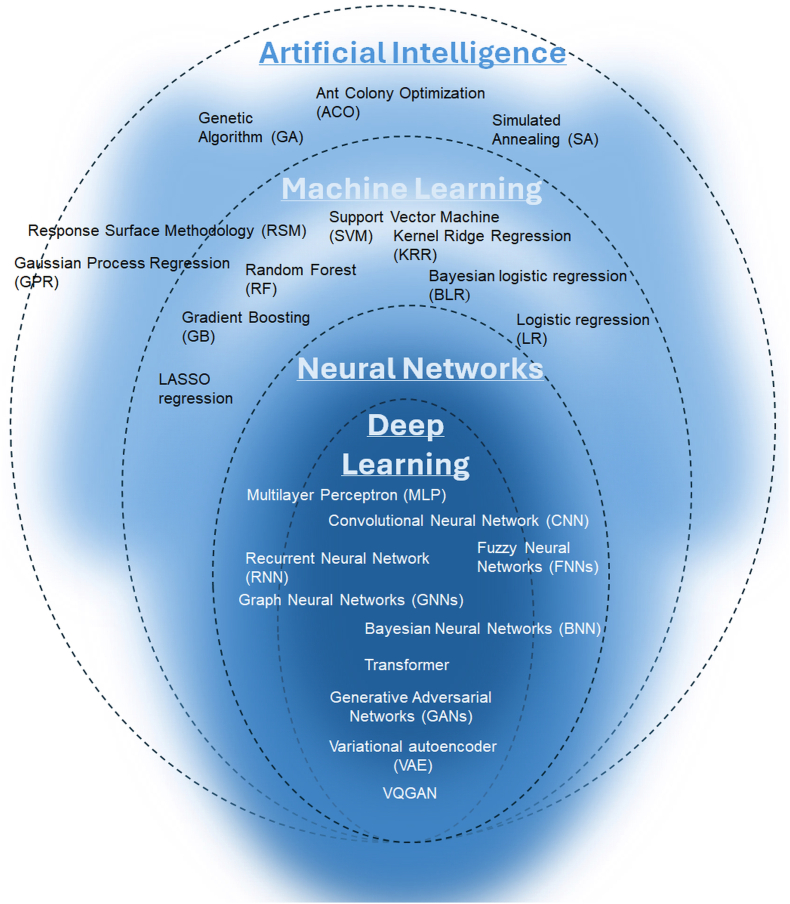
Hierarchical taxonomy of AI methods. AI is organized into nested categories, including machine learning, neural networks, and deep learning, with representative algorithms illustrated at each level.

One of the most popular taxonomies is the hierarchical taxonomy based on the level of complexity and autonomy of the investigated algorithms. A schematic representation of this taxonomy of AI methods is shown in Fig. 6. It should be noted, however, that the boundaries between the delineated areas remain fluid.

The group of methods that are on the borderline between AI and non-AI includes a whole range of tools used in optimization, classification, pattern recognition, and as surrogate models. Good examples of such methods are commonly used regression models, such as Response Surface Methodology (RSM) or Kriging (Gaussian Process Regression). The former is traditionally a statistical design-of-experiments tool, but in modern workflows it is sometimes hybridized with AI (e.g., surrogate models trained with ML), while the latter was widely adopted for Bayesian learning and surrogate modeling. Another group of methods that fall into the so-called “gray zone” includes certain optimization techniques, such as Bayesian Optimization and various metaheuristics. Metaheuristics deserve particular attention, as they are frequently combined with other AI methods, most notably for tuning ML model hyperparameters. Traditionally, metaheuristics are classified as AI methods, even though they neither learn from input data nor autonomously generalize knowledge. Instead, they operate intelligently in a limited sense, searching for good solutions within a defined problem space. Most metaheuristics are inspired by biological processes, such as animal foraging or biological evolution, and are widely applied to diverse optimization problems. Their main advantages are robustness against local optima and effectiveness in optimizing processes for which no complete mathematical description is available. Among the best-known metaheuristics are genetic algorithms, ant colony optimization [212,213], and simulated annealing. Since these methods share strong structural similarities, we focus here on genetic algorithms, as they are the most representative and widely used in materials engineering and drug design.

The genetic algorithm (GA) is a metaheuristic inspired by the principles of natural evolution [214,215]. In this approach, each individual represents a candidate solution, encoded as a chromosome composed of genes corresponding to problem variables. The algorithm begins with a randomly generated population, and a fitness function evaluates the quality of each individual. Fitter individuals are assigned a higher probability of being selected to create the next generation. New offspring are generated through evolutionary operators-crossover (recombination of parent solutions) and mutation (random alterations of genes)-while maintaining a constant population size. This evolutionary cycle is repeated until a predefined termination criterion is satisfied. Although the mathematical proof of the effectiveness of such methods remains incomplete, genetic algorithms are widely regarded as powerful and versatile optimization tools.

Methods classified as **machine learning (ML)** occupy the next level in the taxonomic hierarchy and represent the most relevant and extensive area of interest for AI researchers. ML is a branch of AI concerned with algorithms that improve automatically through experience or exposure to data. These algorithms construct mathematical models from sample data to make predictions or decisions without being explicitly programmed by humans to do so.

**Deep learning** is a subfield of ML that focuses on building and training deep neural networks (DNNs)-networks composed of multiple layers of artificial neurons. These techniques have been particularly successful in applications such as automatic speech recognition, image classification, and natural language processing (NLP). DNNs are described as “deep” because their architecture consists of many layers, each transforming the input data into increasingly abstract representations. In simple neural networks, specific layers can be manually designed to detect certain features, and learning involves adjusting the weights of the connections between neurons. In contrast, large deep networks learn these feature hierarchies automatically. Rather than being explicitly programmed to detect particular features, they infer them from labeled datasets during training. While specialists must still design the overall architecture and prepare the training data, the network itself discovers and represents relevant features. This enables the processing of vast amounts of data and allows the network to automatically learn higher-level abstractions, ultimately capturing complex patterns in the input.

#### 3.1.2 Learning approach taxonomy

A hierarchical taxonomy based on the degree of sophistication-reflected in the level of autonomy exhibited by different algorithms-is not the only way to classify AI methods. Another important criterion is the type of learning approach employed. From this perspective, AI methods can be divided into three main categories: supervised learning, unsupervised learning and reinforcement learning.

The key difference between the AI learning approaches lies in how the training data is prepared. In **supervised learning**, the model is trained on labeled data, meaning that each data point must usually be annotated in advance by a human operator. **Active learning** is a subset of supervised learning, in which the model selectively queries for labels on the most informative data points, thereby reducing reliance on labeling quality without losing learning performance.

By contrast, in **unsupervised learning**, the model is trained on unlabeled data and seeks to uncover underlying structure without explicit labels or guidance. Supervised learning is typically applied to tasks such as object classification and regression/prediction, whereas unsupervised learning is used for clustering or dimensionality reduction, representation learning and image processing.

Beyond these two categories, several hybrid approaches exist. **Semi-supervised learning** leverages a mix of labeled and unlabeled data, making it particularly valuable when labeling is costly or time-consuming. **Self-supervised learning** goes a step further by generating its own labels from raw data, thereby reducing the need for manual annotation.

Another important paradigm is **reinforcement learning**, where a model interacts with its environment and learns to maximize cumulative rewards through trial and error.

### 3.2 Machine learning methods in bioactive materials science

This article focuses primarily on ML methods, as the literature survey consistently indicates that they are the most commonly applied AI approaches in research on bioactive materials. For clarity, the ML methods discussed here are divided into two groups. The first group encompasses methods that are not based on artificial neural networks (ANNs), some of which are of primarily historical importance. The second group comprises neural network-based methods, which represent a much broader and rapidly developing category with strong prospects for applications in bioactive materials research.

#### 3.2.1 Non-neural network approaches

Non-neural network approaches constitute a foundational component of data-driven bioactive materials design, particularly in research settings characterized by limited experimental datasets, the need for interpretability and regulatory transparency. Unlike neural models, these methods often provide clearer relationships between material descriptors and biological outcomes, which is critical for mechanistic understanding and translational applications. Non-neural network methods can be organized into several complementary categories collectively support multiple stages of the bioactive materials design workflow-from early screening and mechanistic inference to nonlinear prediction and adaptive optimization-thereby forming an essential methodological backbone for rational bioactive materials development.

##### 3.2.1.1 Probabilistic classification

Probability-based models, such as logistic regression (LR) and Bayesian logistic regression, are particularly suited for modeling binary or threshold-dependent outcomes, including cytotoxicity, inflammatory response, therapeutic effectiveness and other material responses. By providing explicit probability estimates and interpretable parameter relationships, these methods support risk-aware decision-making and facilitate the identification of material features associated with favorable biological responses.

LR is a supervised ML method used for binary classification (i.e. yes/no). Instead of predicting a continuous value (like linear regression), it predicts the probability that an observation belongs to a certain class. LR models the probability of an outcome using the logistic (sigmoid) function, turning a linear predictor into values between 0 and 1.

LR can predict categorical responses such as cytotoxicity, therapeutic efficacy, or immunological compatibility based on material descriptors. Moreover, the interpretability of LR facilitates mechanism-oriented analysis, allowing the identification of key material features that govern biological performance.

Bayesian logistic regression (BLR) is a statistical modeling approach for binary outcomes that integrates LR with Bayesian inference. By incorporating prior knowledge through prior distributions, BLR enables the formal inclusion of expert beliefs or previous evidence, which are then updated with observed data to obtain posterior parameter estimates. Naïve Bayes is a probabilistic classification method that applies Bayesian theorem with a simplifying assumption of feature independence, making it computationally efficient and effective in many real-world applications.

##### 3.2.1.2 Linear and sparse modeling of structure-properties relationships

Bioactive materials are often characterized by correlated chemical, structural, and mechanical descriptors that jointly influence biological performance. Linear multivariate methods, including Partial Least Squares (PLS) and Least Absolute Shrinkage and Selection Operator (LASSO) regression, enable the modeling of material relationships while addressing multicollinearity and feature redundancy. PLS facilitates dimensionality reduction in highly correlated descriptor spaces, whereas LASSO promotes sparse solutions that highlight key material parameters governing biological outcomes.

PLS regression is a statistical method used in the case of a high number of highly correlated predictors. PLS works by extracting latent components that maximize both the variance in the predictors and their covariance with the response. Unlike the ordinary least squares method, which struggles with multicollinearity, PLS can handle correlated predictors. Additionally, it can be used where predictors outnumber observations.

LASSO regression is a form of linear regression that simultaneously performs prediction and feature selection [214]. By applying a regularization constraint, LASSO can reduce the number of input variables by shrinking certain regression coefficients to zero, thereby effectively eliminating irrelevant predictors. For instance, when predicting a target variable such as material strength from multiple potential factors (e.g., composition, temperature, pressure), some variables may not significantly contribute to the outcome. LASSO addresses this by penalizing less important variables, forcing their coefficients to zero and retaining only the most relevant predictors. This is achieved by augmenting the standard linear regression cost function with an additional penalty term based on the absolute values of the coefficients.

##### 3.2.1.3 Nonlinear and kernel-based learning for materials features prediction

The responses of bioactive materials are usually inherently nonlinear and influenced by complex interactions among compositional, morphological, and mechanical properties. Nonlinear and kernel-based learning approaches - including Gaussian Process Regression (GPR), Support Vector Machines (SVM), Kernel Ridge Regression (KRR), and ensemble tree-based models - are widely employed to capture such interactions. These methods enable robust prediction of continuous or categorical outcomes, such as cell proliferation, differentiation or drug release behavior. In particular, GPR provides uncertainty-aware predictions valuable for risk-sensitive design, while ensemble tree-based models offer feature importance measures that inform material optimization.

GPR is a supervised learning algorithm employed for both regression and classification tasks [216,217]. It enables the prediction of functions based on a set of known data points, while also providing an estimate of the uncertainty associated with these predictions. GPR models the relationship between dependent and independent variables using stochastic processes. As a non-parametric and probabilistic method, it does not impose a predefined functional form; instead, it operates over probability distributions of functions.

When the underlying function exhibits smooth behavior, it can be represented as a Gaussian Process (GP), which defines a probability distribution over possible approximating functions. To construct such a model, training data consisting of input-output pairs must be provided.

A key advantage of GPR is its ability to provide not only point estimates but also associated confidence intervals, offering insights into the reliability of predictions. However, while GPR performs effectively on relatively small datasets, its computational complexity increases significantly with the size of the training set.

GPR plays an important role in the process-oriented design of bioactive materials, particularly in scenarios characterized by limited experimental data and high biological variability. GPR has been widely used to model structure-bioactivity relationships, linking material descriptors to biological outcomes. Moreover, GPR is frequently employed as a surrogate model for computationally expensive finite element analysis or biological assays, enabling efficient exploration of the design space.

SVM is a supervised learning algorithm used for both classification and regression tasks. It is deterministic and works by finding the optimal separating hyperplane that divides data points from different classes. The optimal hyperplane is the one that maximizes the margin, i.e., the distance between the hyperplane and the nearest data points from each class (the support vectors). For linearly separable data, SVM identifies a straight line (in 2D), a plane (in 3D), or a hyperplane (in higher dimensions) that separates classes with the widest margin. When data are not linearly separable, SVM applies the kernel trick, mapping inputs into a higher-dimensional feature space (e.g., using a polynomial kernel) where a linear separation becomes possible. Another method that employs the kernel trick is KRR, which is more commonly applied to regression tasks. Sometimes, before applying SVM, it is beneficial to reduce the dimensionality of the data. Principal Component Analysis (PCA) is most commonly used for this purpose.

SVMs play an important role in classification and early-stage decision making. SVMs are commonly applied to screen candidate materials based on categorical biological outcomes, using limited experimental datasets.

Ensemble tree-based learning methods are ML techniques that combine multiple individual models, each producing only partially accurate predictions, to produce a single, more accurate and robust predictive model than any individual model alone. There are two distinct approaches to the Ensemble learning-Bagging and Boosting. Bagging reduces variance by averaging independent models. A good example of this approach is the Random Forest (RF) method.

Random Forest regression (RFR) is a ML method used to predict continuous values (e.g., temperature, strength, or cost) by combining the predictions of many decision trees [212,213]. RF builds many decision trees, each trained on a slightly different set of data, and averages their predictions to make a strong and stable prediction. The RF can handle nonlinear relationships and rank feature importance. It can be robust to noise and missing values, however at the same time it is less interpretable than a single tree, relatively slower for very large datasets and does not extrapolate well beyond the range of data.

An alternative approach within ensemble tree-based learning is Boosting, which reduces systematic error (bias) by sequentially improving models. A representative example is Gradient Boosting (GB), a ML technique that builds predictive models in stages, typically using decision trees as base learners. In this method, each new model (called a weak learner) is trained to correct the errors (residuals) of the previous models. Gradient descent is applied to a chosen loss function to determine how new learners are added. By combining many weak learners, GB produces a strong and accurate predictive model. GB is a specific implementation of Additive Regression (AR), a general framework in which predictions are generated by combining the contributions of multiple models.

These methods are particularly effective in capturing nonlinear interactions among material descriptors. Owing to their robustness to noise and heterogeneous datasets, ensemble tree-based models are well suited for integrating experimental data from diverse assays. Moreover, feature importance analysis derived from these models provides actionable insights into key material parameters governing material performance.

##### 3.2.1.4 Symbolic and rule-based inference of materials behavior

Beyond predictive accuracy, the development of bioactive materials requires mechanistic understanding of how material features influence biological and other important processes. Symbolic learning approaches, such as Logical Analysis of Data (LAD) and Inductive Logic Programming (ILP), focus on extracting human-interpretable rules that describe relationships between material characteristics and responses. By incorporating prior knowledge and generating explicit logical patterns, these methods support hypothesis generation and mechanism-oriented reasoning. In a process-oriented design context, symbolic approaches complement statistical models by providing interpretable insights that guide targeted experimental validation and theory development.

LAD is a combinatorial and logical approach to data analysis and classification. It is particularly suited for binary or categorical data. Because LAD produces rules in logical form, it is highly interpretable compared to many black-box models. Unlike statistical methods, LAD does not rely on probabilistic assumptions. LAD detects patterns based on input variables or predictors (such as molecular descriptors) and provides predictions as either positive or negative outcomes.

ILP is an ML approach that combines techniques from logic programming and inductive inference. It learns general rules from examples. ILP uses heuristic search to infer hypotheses from observed cases, producing a set of logical rules that, in the case of bioactive materials can link material characteristics-such as chemical functionality, surface structure, or degradation behavior-to biological responses including cell adhesion, differentiation, or immunomodulation.

##### 3.2.1.5 Closed-loop optimization and inverse materials design

The ultimate objective of bioactive materials development is the rational identification of material formulations that achieve predefined targets. Sequential Model-Based Optimization (SMBO) provides a systematic framework for iterative design by coupling predictive models with adaptive sampling strategies. By proposing new material candidates based on predicted performance and associated uncertainty, SMBO enables efficient exploration of high-dimensional design spaces while minimizing experimental cost. Integrated within a closed-loop workflow, this approach facilitates inverse materials design and accelerates the translation from computational prediction to experimental validation.

SMBO provides a general approach to optimizing functions that are expensive to evaluate, lack an explicit analytical form (black-box functions), and may be noisy. This class of optimization relies on surrogate models that approximate the target function. By doing so, surrogate models enable efficient estimation of function values at points selected by the SMBO algorithm. The process is both sequential-evaluating one sample at a time-and model-based-using the surrogate model to guide the search. One of the most widely used SMBO techniques is Bayesian Optimization. In this approach, the surrogate model is typically GP, and the selection of the next evaluation point is guided by Bayesian acquisition functions.

#### 3.2.2 AI approaches based on neural networks

Despite their numerous advantages, non-neural network AI approaches also present several important limitations. One of the most significant challenges is their limited ability to handle high-dimensional and unstructured data, such as microscopic images, omics datasets, and complex three-dimensional structural representations. These methods typically rely on predefined material descriptors and often require prior feature engineering and dimensionality reduction, which may introduce bias or result in information loss. In addition, many non-neural approaches become computationally expensive when applied to large-scale datasets or complex optimization problems.

**Fig. 7:**
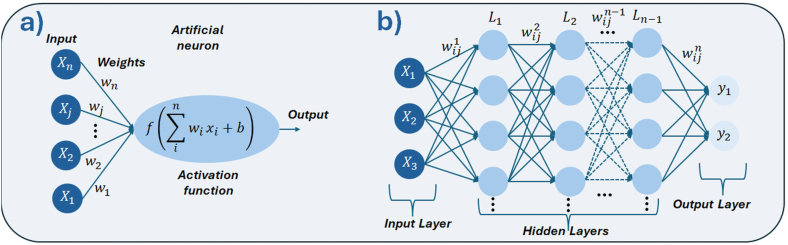
Structure of a single artificial neuron and artificial neural network: a) Scheme of artificial neuron, showing inputs, weights, summation, activation function, and output. b) Scheme of the most fundamental ANN architecture-multilayer perceptron [218].

ANN-based methods have emerged as a powerful alternative to address many of these challenges. Inspired by the structure and functioning of biological neural systems, neural networks can learn complex feature representations directly from raw data. This capacity makes them particularly suitable for modeling high-dimensional, nonlinear, and multimodal datasets commonly encountered in bioactive materials research. As with their biological counterparts, the fundamental building block of an ANN is the neuron. Neural networks typically consist of many interconnected neurons organized into layers (Fig. 7).

The most fundamental type of ANN, Multilayer Perceptron (MLP), is composed of an input layer, one or more hidden layers, and an output layer. The input layer receives data (e.g., numerical values), the hidden layers process this data through weighted connections combined with activation functions, and the output layer produces the result (e.g., a classification or a prediction). For an ANN to function as a predictive or classification tool, it must first undergo training, a process to determine all weights that interconnect neurons. Training data-often obtained from experiments or observations-are provided as inputs to the network. The predicted output is then compared to the expected (true) output, and the difference (error) is quantified. This error is propagated backward through the network in a process known as backpropagation, which updates the weights of the connections, typically using an optimization algorithm such as gradient descent. The process is repeated iteratively until the network achieves satisfactory predictive accuracy and the neural network weights are then finalized.

MLP is a type of feedforward neural network, meaning that data flows only in one direction-from input to output. Despite its simplicity, the MLP remains widely used for both classification and regression tasks, particularly in applications involving structured (tabular) data. The MLP constitutes the foundational architecture for many advanced neural network systems, which can be grouped into the core categories presented in Table 2. This classification is primarily based on the type of data being processed, namely spatial data, sequential data, and graph-structured data. In addition, separate categories are distinguished for generative neural networks and for models explicitly designed to account for uncertainty, whether arising from inherent variability in the data (aleatoric uncertainty) or from limitations of the modeling process itself (epistemic uncertainty). Although this classification is, to some extent, arbitrary, most neural network models can be assigned to one of the core categories presented below. Moreover, this framework reflects the specific needs of materials engineering, facilitating the selection of an appropriate network architecture tailored to experimental data.

**Table 2:** Core categories of artificial neural networks.

Network Type	Description	Examples	Ref.
ANNs for spatial data processing-mainly based on CNN (Convolutional Neural Network)	Captures spatial/local patterns (e.g., images); Used for image classification, medical image analysis, analysis of scanning electron microscopy (SEM) images, confocal microscopy images, histological sections, and micro-CT data	CNN, ResNet, ResNeXt, Xception, U-Net	[219,220]
ANNs for sequential data analysis and processing-mainly based on RNN (Recurrent Neural Network)	Processes sequential/time-dependent data, i.e., information collected over time or ordered stages, where observations are not independent and the current states depend on previous states	RNN, LSTM, ConvLSTM, Transformer	[[221], [222], [223], [224]]
ANNs for graph-structured data analysis and processing-mainly based on GNN (Graph Neural Network)	Works with graph-structured data; analysis of the relational systems, i.e. atoms connected by chemical bonds, polymer chains forming networks, pores connected in a tissue engineering scaffold.	GNN, GCN, GAT, GraphSAGE, PNA	[[225], [226], [227], [228]]
Generative models	Designed to learn the underlying data distribution and generate new data samples that closely resemble it.	GAN, VAE, cVAE, VQGAN, DM, U-Net	[[229], [230], [231], [232], [233], [234]]
Uncertainty-aware neural models	Quantifies probabilistic uncertainty of prediction; addresses linguistic vagueness, which refers to ambiguity in definitions, imprecise boundaries between categories, or incomplete information	BNN, FNNs	[235,236]

In the following sections, each of the neural network categories listed in Table 2 is described in detail. Furthermore, architectures that build upon these foundational concepts are introduced, with particular emphasis on those applied in bioactive materials engineering.

##### 3.2.2.1 Spatial data analysis and processing

Bioactive materials research heavily relies on the imaging and analysis of spatial data from various sources. These include scanning electron microscopy (SEM) images of material microarchitecture, confocal microscopy images, histological sections, and micro-CT data characterizing porosity and phase distribution. Specialized AI-based tools are therefore required to automatically learn relevant spatial features, capture complex texture and morphological patterns, and detect subtle spatial correlations that are not easily quantified using conventional analysis methods.

**Fig. 8:**
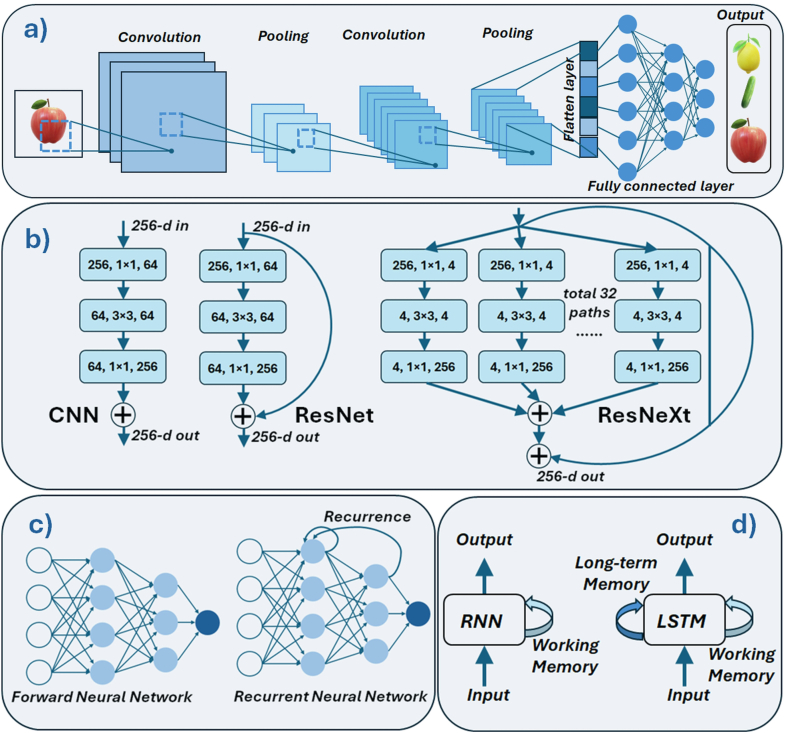
Overview of neural models for sequential data analysis and processing: a) working principle of CNN [218]; b) schematic illustration presenting structural difference between CNN, ResNet, and ResNeXt architectures (ResNeXt with cardinality of 32) [237]; c) difference between RNN and classic neural model-RNNs have recurrent connections that enable information to persist across time steps [238]; d) difference between RNN and LSTM-LSTMs have special memory cells and gates that let them learn long-term dependencies in sequential data [239].

Convolutional Neural Network (CNN) is a specialized type of ANN designed for the processing of image and spatial data. CNNs are particularly effective in tasks such as image classification [219], object detection, and medical image analysis. In these networks, raw image data-typically represented as a matrix-is fed into the input layer (Fig. 8a). The data is then passed to the convolutional layer, where key features such as edges and textures are detected. Within the convolutional layer, filters (also represented as matrices of smaller dimensions than the input image) slide over the image and perform element-wise multiplications-a mathematical operation known as convolution. This process generates a set of feature maps, which retain spatial relationships and highlight significant image features.

Subsequently, the data is passed through pooling layers, which reduce the spatial dimensions of the feature maps while preserving the most important information. After several convolutional and pooling operations, the resulting feature maps are flattened into a one-dimensional vector. This vector is then processed through one or more fully connected layers, similar to those found in traditional neural networks. These layers perform high-level reasoning and ultimately generate the network's final output, such as a class score. One subcategory of CNNs that has been used successfully in materials engineering is Residual Networks (ResNets) (Fig. 8b) [220]. They are deep feedforward CNNs that use so-called residual connections schematically presented in Fig. 8b. ResNet became appreciated because it enabled training of extremely DNNs (over 100 layers), which was previously very difficult due to problems like the exploding gradients and degradation (accuracy decreasing when adding more layers).

ResNeXt is an improved version of ResNet, introduced in 2017. It keeps the residual block idea from ResNet but introduces a new dimension: cardinality, which is the number of parallel paths inside a block (Fig. 8b). In ResNeXt, each block has multiple parallel paths of the same topology, but with different weights, and the outputs are summed. ResNeXt achieves higher accuracy with fewer parameters compared to ResNet. For the same computational budget, increasing cardinality is more effective than just making the network deeper or wider.

Xception is a CNN architecture proposed by François Chollet in 2017. Unlike conventional CNNs, which combine spatial and channel information in a single operation, Xception factorizes this process into two steps: depthwise convolution (capturing spatial features independently per channel) and pointwise convolution (modeling cross-channel relationships). Each channel represents some features of the processed image. This design improves computational efficiency and often enhances accuracy compared to traditional CNN architectures.

Another popular subcategory of CNNs is U-Net. Its architecture follows a symmetric encoder-decoder structure, where the encoder consists of multiple convolutional layers used to extract features from the input image, and the decoder consists of another set of convolutional layers used to reconstruct a pixel-wise segmentation image from the encoded feature map. A key feature of U-Net is its skip connections which directly transfer feature maps from the encoder to the decoder, preserving fine-grained spatial information. U-Net works very well with limited training data and shows strong generalization ability, making it particularly effective for biomedical applications such as cell and tissue segmentation.

##### 3.2.2.2 Sequential data analysis and processing

Sequential data refers to information collected over time or ordered stages, where observations are not independent and the current states depend on previous states. In the context of bioactive materials research, sequential data typically arise from material degradation over time, drug release kinetics or cell-material interaction dynamics. All the mentioned processes are inherently time-dependent, making sequential modeling essential.

Recurrent Neural Network (RNN) is type of neural network being particularly well-suited for processing sequential data, in which the order of elements is especially important [221]. The most popular examples include time series, speech, text, and biological signals (e.g., electrocardiogram and electroencephalogram signals). Unlike standard ANNs, RNNs have recurrent connections that enable information to persist across time steps (Fig. 8c). This means that the state of the network at a given moment depends not only on the current input but also, in part, on inputs from the past. RNNs are well suited for capturing temporal dependencies, such as predicting the next word in a sentence or forecasting future values in a time series. Notably, RNNs could be a powerful tool in materials engineering, especially for modeling phenomena where the state of a material depends on its past history [222,223].

Unlike basic RNNs, which often forget long-term information due to the vanishing/exploding gradient problem, Long Short-Term Memory networks (LSTMs) can remember information for long periods of time (Fig. 8d). They have special memory cells and gates that learn long-term dependencies in sequential data. Convolutional LSTM (ConvLSTM) is a specific combination of convolutional networks and LSTM. It is a type of network that is particularly useful for analyzing and predicting spatiotemporal data. In the case of ConvLSTM networks, fully connected operations characteristic of classic LSTMs are replaced with convolutions, which preserve spatial relationships.

While RNNs and their variants like LSTMs have been highly effective for modelling sequential data, they struggle with long-range dependencies and slow, sequential processing due to their inherently sequential computations. Transformers were initially proposed [224] to overcome these challenges by replacing the step-by-step processing of RNNs with a parallelizable self-attention mechanism. This has made transformers significantly efficient to capture dependencies across arbitrary distances while enabling much faster training.

##### 3.2.2.3 Graph-structured data analysis

In bioactive materials research, many systems are inherently relational. Good exemplifications are atoms connected by chemical bonds, polymer chains forming networks, pores connected in a tissue engineering scaffold. Graph Neural Networks (GNNs) are a class of deep learning models, which allow learning directly from these relational structures without converting them into simplified descriptors. They are specifically designed to work with graph-structured data. Unlike traditional neural networks that operate on regularly structured data (like images or sequences), GNNs are built to handle non-Euclidean data where elements (nodes) are connected in structurally arbitrary ways via edges. A graph is a data structure consisting of nodes (also called vertices) that are interconnected by edges, and GNN learns to estimate these edges. Graphs are used to model many real-world systems, such as social networks, molecular structures [225], transportation systems, knowledge graphs, and biological networks. The central idea behind GNNs is to enable each node in a graph to learn from its neighborhood-that is, to aggregate information from the nodes it is connected to. This is typically done through an iterative process called message passing or neighborhood aggregation, which allow information to propagate across the graph.

There are several types of GNN architectures, each with slightly different mechanisms for aggregation. Examples include Graph Convolutional Networks (GCNs), which generalize the concept of convolution from grids (like in images) to graphs [226,227]; Graph Attention Networks (GATs), which use attention mechanisms to weight the importance of neighboring nodes [228]; and GraphSAGE, which introduces efficient neighborhood sampling for large graphs.

One of the strengths of GNNs is their ability to learn useful representations of graph components-such as nodes, edges, or entire graphs-that can be used for a wide range of tasks. For instance, node classification involves predicting a label for each node, link prediction infers missing edges, and graph classification assigns a label to the entire graph (e.g., determining whether a molecule is toxic or not). However, GNNs are not without limitations. They tend to focus on local neighborhoods, and capturing long-range dependencies across a graph can be difficult, especially in deep architectures where issues like over-smoothing and vanishing gradients may arise. Additionally, scalability can be a challenge for very large graphs, though methods like sampling, clustering, and distributed training have been developed to address this.

Principal Neighborhood Aggregation (PNA) is a kind of GNN architecture designed to improve the way in which the information is aggregated from neighboring nodes in a graph [240]-especially in heterogeneous or structurally diverse graphs, such as molecules or materials, where each atom aggregates information from its neighboring atoms. PNA enhances this aggregation step by making it more informative than standard GNNs. Standard GNNs (like GCNs or GATs) usually use simple aggregators, such as the mean of neighbor features, which can struggle to capture complex patterns-especially when neighbor counts vary significantly or when node features are noisy or highly diverse. In contrast to standard GNN, PNA combines different functions (mean, max, min, std, etc.) and additionally can adjust aggregation based on the number of neighbors a node has. This captures richer information about both the local structure and features. PNA could be especially useful in materials science for modeling graph-based structure -property relationships.

##### 3.2.2.4 Generative models in materials science

Generative models are one of the most important classes of AI models. Unlike traditional models that focus mainly on recognition or classification, generative AI (Gen-AI) models are designed to learn the underlying data distribution and generate new data samples that closely resemble it. Generative AI models are widely used in domain adaptation to reduce discrepancies between source and target domains by generating synthetic data or aligning feature distributions. The following sections highlight the generative models most widely applied in materials science and engineering, with particular emphasis on the analysis and design of bioactive materials.

**Fig. 9:**
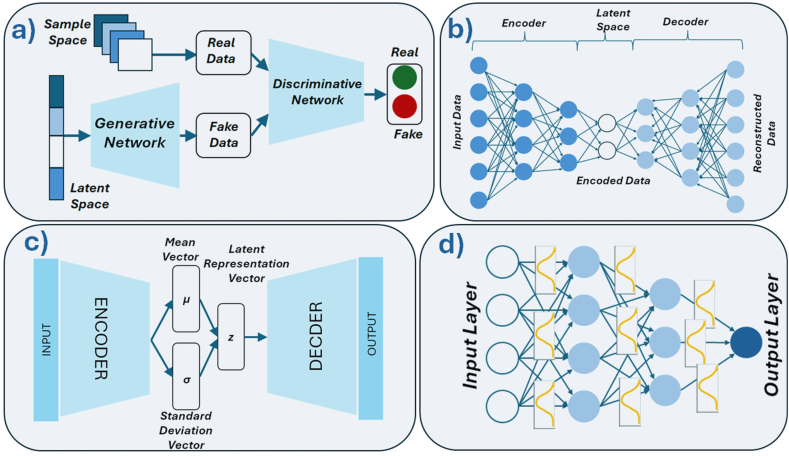
Overview of generative and Bayesian neural network architectures: a) architecture of GAN [229]; b) standard autoencoder neural network [241]; c) architecture of variational autoencoder [242]; d) Bayesian neural network [235]-unlike ANNs with fixed weights, BNNs learn weight distributions and generate output distributions, enabling quantitative uncertainty estimation.

Generative Adversarial Networks (GANs) are a class of ML models capable of generating synthetic data-such as realistic images, audio, or molecular structures-that closely resemble real-world data. A GAN consists of two key components, the generator and discriminator, that are trained simultaneously in a competitive framework (Fig. 9a). The generator aims to produce data that mimics real examples (e.g., synthetic images, acoustic waveforms, or molecular geometries), while the discriminator attempts to distinguish between genuine and generated data, classifying inputs as either real or fake. During training, the generator iteratively improves its ability to deceive the discriminator, while the discriminator refines its capacity to detect synthetic outputs. Over time, the generator can produce outputs that are asymptotically indistinguishable from empirical authentic data. In materials science and engineering, GANs can be applied to generate novel crystal structures, microstructures, or molecular configurations, thereby facilitating accelerated materials discovery and design [229,230]. However, GANs suffer from training instability that leads to poor results and require significant fine tuning of the model parameters. Additionally, their requirement of high computing power limits scalability for complex scientific problems.

While GANs generate new data by framing a competitive game between two networks, variational autoencoders (VAEs) take a probabilistic approach, learning a latent representation from which new samples are generated in a controlled manner. This approach provides complementary strengths to GANs in modeling structured data, while offering a mathematically principled framework for data generation. A VAE consists of three main components: encoder, latent space and decoder (Fig. 9b). The encoder maps the input data into a lower-dimensional representation vector [231,232]. This is known as the latent space. The decoder's task is to transform the data from latent space to a reconstructed form (with the dimension of the input vector). A key feature of VAE is that instead of encoding input into a fixed latent vector (Fig. 9c), the encoder learns to encode the input as a probability distribution (usually a Gaussian) in the latent space. This makes it possible for the decoder to sample from the learned latent distribution to generate new, previously unseen data that arises based on learned patterns. This type of AI generative model could be used for image generation, data compression as well as molecules and drug design. There is also a VAE variant that allows for some limited control over the content generated by VAE. This is called a conditional variational autoencoder (cVAE). cVAE algorithms have been successfully used for antibacterial peptide generation [233,234].

Vector Quantized Generative Adversarial Network (VQGAN) is a type of generative model that combines two ideas: Vector Quantized Variational Autoencoder (VQ-VAE) and GAN, with Transformer built on top for sequence modelling. VQGAN encodes an image into a discrete latent space. Instead of continuous embeddings, it uses so-called codebook of visual tokens. Codebook is a finite set of learned vectors (embeddings) that act like a catalogue of building blocks for representing data. Each token is a learned embedding that represents a small patch or feature of the data, and the data is effectively represented as a sequence of these tokens. Instead of representing data in a continuous high-dimensional space, the model maps it to the nearest code in the codebook and quantizes the token. This process is called vector quantization. The GAN's adversarial training allows VQGAN to generate sharp, high-resolution images, while the discrete token representation makes it compatible with sequence-based models such as Transformers. VQGAN has been applied for text-to-image generation, image compression as well as for creative AI art.

Compared with VQGAN, Diffusion Model (DM) takes a fundamentally different approach to generate new data-such as images, audio, video, or protein sequences-by first adding random noise to a training dataset through a series of diffusion steps and then learning to reverse this process. During the training, a neural network is trained to gradually denoise the noisy training set step by step to construct the desired outputs. Unlike VAEs, the latent space of DMs has the same dimensionality as the original data. DMs use a neural network to learn the denoising process. To generate new data, the process begins with a field of random noise, which is iteratively refined by the trained network until a high-quality output is produced. Various architectures, such as Transformers (in the case of attention-based DMs) or VAEs, can be employed for the denoising task. One of the most widely used approaches is the U-Net, a type of autoencoder that combines CNNs with an encoder-decoder structure to efficiently perform stepwise denoising. This stepwise process enables DM to generate highly detailed and diverse samples without relying on a discrete latent space, complementing the strengths and limitations of VAE and VQGAN. DM has been widely used in image and video generation, protein design, computational biomedical imaging, etc.

##### 3.2.2.5 Uncertainty-aware neural modeling

Bioactive material systems, like all physical systems, are characterized by some variability. Usually, researchers could use limited experimental datasets, and all the measurements are to some extent noisy. In this context, deterministic prediction is insufficient. That is why uncertainty-aware neural models could be very useful. Bayesian Neural Networks (BNN) are a type of neural network that quantifies prediction uncertainty. It combines the advantages of traditional ANNs with the possibility of Bayesian inference (Fig. 9d). Instead of learning fixed weights, like ANN, BNN learns distributions over weights. During prediction, it samples from these distributions multiple times, giving a distribution over outputs. This allows for a quantitative assessment of the reliability of predictions.

Another form of uncertainty arises from linguistic vagueness, which refers to ambiguity in definitions, imprecise boundaries between categories, or incomplete information. This type of uncertainty is not probabilistic in nature but stems from the gradual and often subjective interpretation of concepts (e.g., “high porosity” or “moderate degradation rate”). It can be effectively addressed using fuzzy logic systems and neuro-fuzzy models. Neuro-fuzzy systems combine the strengths of neural networks and fuzzy logic. In the literature, they are often referred to as Fuzzy Neural Networks (FNNs). Generally, neural networks excel at pattern recognition, while fuzzy logic is well-suited for handling imprecise or incomplete information. Unlike traditional ANNs, FNNs are well interpretable. They consist of two components: a fuzzy logic part that represents knowledge through human-readable IF–THEN rules, and a neural network part that learns from data by automatically adjusting membership functions and refining rules, similar to how weights are updated in a neural network. This enables FNNs to learn effectively from data while keeping the system transparent and understandable in human terms.

### 3.3 AI-based quantitative structure-activity relationship (QSAR) tools

Quantitative Structure-Property Relationship (QSPR) and Quantitative Structure-Activity Relationships (QSAR) are computational methods that model the relationship between the chemical structure of a given molecule and its physicochemical (QSPR) or biological (QSAR) properties using mathematical and statistical tools. The level of complexity of the problem presented makes ML algorithms an ideal candidate for the QSAR tools. It was proven that once trained, the AI models can effectively predict the properties of a given molecule from its structure alone.

#### 3.3.1 QSAR data organization

Regardless of the type of AI prediction tool used, QSPR/QSAR models often require the calculation of descriptors (e.g., molecular weight, number of hydrogen bond donors) and fingerprints of molecules from a training set. Input data for QSPR/QSAR models is most often provided in the form of files in *simplified molecular input-line entry system* (SMILES) format. It is a way of coding the chemical structures in the form of plain text. There are a number of applications for calculating descriptors and fingerprints (PaDEL, RDKit, or Dragon). After calculating the descriptors and selecting them, the predictive model is trained. The trained model should be validated using data from outside the training set.

Given that the number of descriptors can be very large, one of the significant problems with QSAR is redundancy, since many sets of descriptors could be collinear. Choosing the right subset of descriptors improves interpretability, reduces overfitting, and increases predictive performance. Therefore, efforts are made to reduce the number of descriptors [243]. Various methods of descriptor selection are used. The most primitive ones are the filter methods. In this case, the criterion for eliminating descriptors can be a variance threshold or a relatively high correlation coefficient. Wrapper methods belong to the group of much more advanced methods. These are usually model-driven methods using metaheuristic algorithms (e.g., genetic algorithms, simulated annealing). The criterion for selecting a given subset of descriptors is its predictive performance. The third group of feature selection methods are the embedded methods. They perform feature selection during model training itself [244]. Unlike filter methods (independent of model) and wrapper methods, embedded methods integrate selection into the learning process by penalizing or weighting descriptors based on their importance. Finally, there are also methods that combine the above-mentioned approaches.

**Fig. 10:**
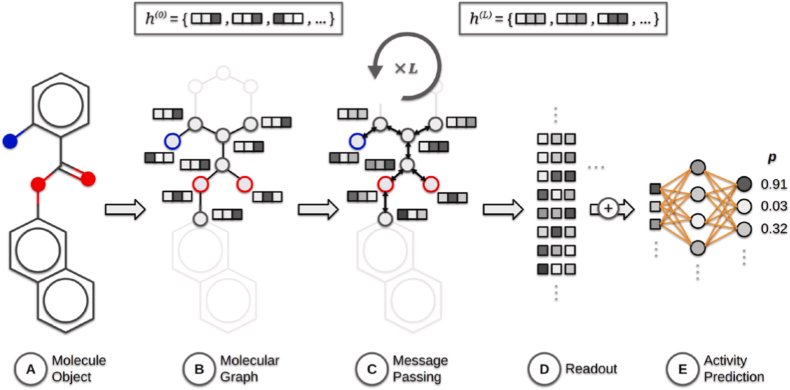
Overview of a QSAR model using GNN. A molecule (a) is transformed into a molecular graph (b) with nodes (atoms), edges (bonds), and features h^(0)^. After L updates (c), features become h^(L)^, which are reduced to a molecular encoding (d) for activity prediction (e). For clarity, edge features and some substructures are omitted [246].

It should be emphasized that modern generative models could operate directly on SMILES strings without explicit calculation of descriptors. GNNs seem to be particularly useful for this task [245]. It is known that the structure of a molecule can be represented as a graph G = (V, E), where V is the set of nodes corresponding to atoms, and E is the set of edges corresponding to bonds [246]. In practice, GNN models used in QSAR are most often constructed as message-passing neural networks (MPNN) (Fig. 10) [246]. One of the key operations in such networks is the so-called message passing. During this operation, each node updates its state by exchanging messages with its neighbors along the edges of the graph. This operation is repeated iteratively in order to aggregate as much information as possible. Each node updates its representation (feature vector) by combining its current state with messages received from neighboring nodes. Since this process is repeated iteratively, each node gradually incorporates information from an increasingly larger part of the graph. If the solved problem is graph-level (e.g., predicting whether a molecule is toxic), all node embeddings need to be combined into a single vector that represents the entire graph. This combining step is called the readout function. The next step is the biological activity prediction using feedforward neural network.

It should be emphasized that well-trained ML models coupled with metaheuristic methods (like genetic algorithms) could be powerful tools capable of designing molecules with specific physicochemical and biological properties [247,248].

#### 3.3.2 Nano-QSAR

Nano-QSAR is a variant of QSAR specifically dedicated to predicting the biological activity of nanoparticles. The concept of nano-QSAR was first presented by Puzyn and co-workers [249]. Nano-QSAR is closely related to the idea of quasi-QSAR. Quasi-QSAR tools allow the prediction of properties of various types of non-molecular objects, i.e., objects that are not traditional chemical compounds, but that can still be described by structured descriptors. Examples of such objects are nanoparticles, polymeric mixtures, as well as environmental factors. To encode non-molecular objects or experimental conditions, quasi-SMILES strings are used, characterized by syntax resembling SMILES used in classical QSAR. A major platform for quasi-QSAR is CORAL software. Initially quasi-QSAR was used for the prediction of mutagenic potential of multi-walled carbon nanotubes (MWCNTs) [250]. There are documented attempts to use AI methods, such as neural networks, in nano-QSAR analysis [251].

### 3.4 Summary of AI tools

Overall, this section innovatively classifies current AI methods using a functional taxonomy specific to bioactive materials and outlines the fundamental principles of each approach. This classification systematically groups different AI methods based on their characteristics and applicability, clarifying their roles in different applications. However, challenges still remain in AI methods, such as handling high-dimensional or noisy data, addressing limited data availability, and reducing computational cost. Additionally, model transparency is still a concern, as many advanced AI methods function as “black boxes”, posing challenges for mechanistic interpretation and regulatory acceptance.

## 4 Applications of AI in bioactive materials

Building on the preceding introduction of bioactive materials and AI tools, this section outlines the applications of AI in bioactive materials. Here, AI models are presented in the context of specific applications, focusing on their functional roles and achieved performance rather than methodological details. In contrast to previous reviews that classify AI applications by material type (e.g., metals, ceramics, and polymers) [[17], [18], [19], [20]], we propose a process-oriented framework aligned with the sequential workflow of material development, from material design and fabrication optimization to property prediction, in vitro assessments, and in vivo assessments. This classification offers a clearer view of how AI contributes at distinct stages of material development, thereby helping to improve understanding and provide guidance for future research of AI in bioactive materials. Table 3 shows the AI methods, materials and applications in exemplary studies during different development processes of bioactive materials.

**Table 3:** AI methods, materials and applications in exemplary studies during different development processes of bioactive materials.

Material development processes		AI methods	Materials	Applications	Ref.
Material design	Material formulations	ANN	Ti alloys	Design compositions of low-modulus Ti alloys	[252]
		GNN	Proteins	Design protein sequences with predefined structural or functional targets	[253]
		DM	Proteins	Design protein sequences with desired mechanical properties	[254]
		Bayesian optimization, GP	Proteins	Design glycoside hydrolase enzymes with high thermostability	[255]
		GPR	Copolymers	Design copolymers to enhance REA of PPHs	[256]
		ILP	Hydrogels	Design hydrogels with enhanced structure fidelity	[257]
		RFR, GP	Hydrogels	Design hydrogels with high adhesive strengths in water	[258]
	Material structures	VQGAN	/	Reconstruct the microstructure of diatoms	[259]
		CNN	/	Design spinodal structures	[260]
Fabrication optimization	3D printing	ANN	PLA	Predict tensile strengths of plastic components manufactured using FDM	[261]
		Neural network	PLA	Analyze mechanical behaviors of FDM-printed lattice-cell structures	[262]
		ResNet 50	PLA	Autonomously monitor and correct FDM printers	[263]
		ResNeXt-50	GelMA hydrogels	Detect anomalies during DIW printing of GelMA hydrogels	[264]
		Bayesian optimization	GelMA and HAMA hydrogels	Optimize DIW printing of GelMA and HAMA bioinks	[265]
		RF	PPF	Predict print qualities of PPF scaffolds fabricated by DIW	[266]
		LASSO regression	Alginate hydrogels	Optimize the high-fidelity 3D printing of alginate-based biopolymers using the FRESH technique	[267]
	Bioprinting	Over 40 AI models	Hydrogels	Predict the quality of bioprinted cell-laden scaffolds	[268]
Property prediction	T_g_	RF	PHA	Predict the T_g_ of PHA homopolymers and copolymers	[269]
		RF	TPU	Predict the T_g_ of linear polyurethane elastomers	[270]
	Mechanical properties	ANN	CAS glasses	Predict Young's modulus of CAS glasses	[271]
		Six supervised ML algorithms	17 materials	Identify and rank suitable biomaterials for bone scaffold applications	[272]
	Solubility	RF, AR	Oxide glasses	Predict dissolution behaviors of biomedical oxide glasses	[273]
		RF	Collagen scaffolds	Quantify the biodegradation of type I collagen scaffolds	[274]
		ANN, KRR	Gelatin hydrogels	Predict degradation rates of genipin-crosslinked gelatin scaffolds	[275]
	Nanozyme activity	DNN	Nanozymes	Predict activities of nanozymes	[276]
	Hydrogel formation	GB	Peptidelike compounds	Predict gelation behaviors of peptidelike compounds	[277]
		LR	Nucleoside derivatives	Predict gelation behaviors of nucleoside derivatives	[278]
	Protein characteristics	End-to-end DL	Proteins	Predict secondary structure contents of proteins	[279]
		GNN	Proteins	Predict protein natural frequencies	[280]
	Protein adsorption	ANN	Polyarylates	Predict the protein adsorption and cellular response to the surfaces of polyarylates	[281]
		QSPR	SAMs	Quantify protein adsorption on functionalized SAMs	[282]
		ANN	SAMs	Predict the water contact angle and protein adsorption on SAMs	[283]
		RF	Nanoparticles	Predict the functional composition of the protein corona and cellular recognition of nanoparticles	[284]
In vitro assessments	Cytotoxicity	QSAR	Metal oxide nanoparticles	Predict the cytotoxicity of 18 metal oxide nanoparticles to HaCaT cells	[285]
		Nano-QSAR	Metal oxide nanoparticles	Predict the cytotoxicity of 18 metal oxide nanoparticles to HaCaT cells	[286]
		Quasi-QSAR	Metal oxide nanomaterials	Predict the viability of BEAS-2B and HaCaT cells exposed to 21 metal oxide nanomaterials	[287]
		PTML	DDNS	Predict the biological activity and toxicity of DDNS	[288]
	Cell adhesion	PLS	Acrylate polymer	Investigate the influence of acrylate polymer surface chemistry on hEB cell adhesion	[289]
		BNN	Acrylate polymer	Predict the adhesion of hEB cells on acrylate polymer surfaces	[290]
		SVM	PCL	Quantify and classify single-cell confinement in melt electrowritten PCL scaffolds	[291]
		RF	Polyacrylamide copolymers	Identify hydrogel coatings capable of resisting platelet adhesion on implantable biosensors	[292]
	Cell differentiation	CNN	Hormones, neurotrophins, nanoparticles	Predict the differentiation of NSCs exposed to diverse inducers	[293]
		GPR	Laminin-coated glasses	Predict the differentiation in NSCs derived from adult and hiPSCs across diverse nanotopographies	[294]
		KNN	Different biomaterials	Predict the differentiation of hMSCs across different biomaterials	[295]
		OCNN	Collagen I, hyaluronic acid, amyloid fibrils, polydopamine	Predict the osteogenic differentiation of rBMSCs	[296]
	Metabolism	DNN	PEGylated AuNPs	Predict the in vivo fate of PEGylated AuNPs	[297]
	Antimicrobial activity	Neurofuzzy logic technology	BG	Predict the antibacterial performance of BG	[236]
	Immune responses	GB regression	Polystyrene	Examine the influence of microscale surface topographies on human monocyte attachment and macrophage differentiation	[298]
		GPR	Glass, silicon, Ti	Study the effect of nanotopographies on macrophage polarization	[299]
		RF, SVM, MLP	(Meth)acrylate and (meth)acrylamide polymers	Identify simple (meth)acrylate and (meth)acrylamide polymers capable of directing macrophage polarization towards different phenotypes	[300]
		BLR, KNN, Naïve Bayes	50 distinct polymers	Predict immunomodulatory properties of anti-inflammatory polymers	[301]
In vivo assessments		CNN	Soft-tissue fillers	Quantify intradermal volume of hydrogels	[302]
		LAD	Poly (β-amino esters)	Investigate structure-activity relationships in polymeric gene delivery	[303]
		Three neural networks	Sr-HT-Gahnite	Study bone regeneration processes within porous tissue scaffolds	[304]

### 4.1 Material design

Material design plays a fundamental role in the development of high-performance bioactive materials, as it directly determines their structural, functional, and biological properties. Traditionally, this process has been driven by empirical knowledge or theoretical frameworks, where candidate materials are explored within a constrained range. While such methods have yielded valuable outcomes, they often overlook unconventional or counterintuitive formulations and structures that may exhibit superior performance. The integration of AI into material design provides a powerful alternative to overcome these limitations. AI-driven design typically begins with training ML models based on large datasets that map the formulations and structures of materials to their properties. Once trained, these models can perform inverse design by predicting or generating optimal materials according to predefined target properties, which may lie beyond the scope of traditional methods and enable the discovery of entirely novel materials with potentially groundbreaking performance. This data-driven approach significantly accelerates innovation in the design of bioactive materials, especially when multiple performance criteria need to be met simultaneously.

#### 4.1.1 Material formulations

Material formulations involve the design of molecular structure, chemical composition, component ratios, mass content, and functional additives to achieve specific properties. Yen and co-workers employed ML to accelerate the design of low-modulus Ti alloys for biomedical implants [252]. A ML framework named “βLow” was developed, which combined two ANNs trained on small experimental datasets to predict Young's modulus and martensitic start temperature from alloying compositions. The models used the weight fractions of biocompatible alloying elements as input features, and generated outputs corresponding to mechanical properties and phase stability. Following ML-based high-throughput screening under constraints of low modulus and limited β stabilizer content, several alloy candidates were recommended. Among them, Ti-12Nb-12Zr-12Sn was experimentally validated, showing low modulus, excellent biocompatibility, and reduced cost. This study revealed that a large portion of the compositional space for low-modulus biomedical Ti alloys remains unexplored, highlighting the value of data-driven alloy design.

In addition to metals, protein sequences have also been designed by AI tools [305]. MaterioFormer, a transformer-GNN, was developed to solve both forward prediction and inverse design in protein modeling [253]. The model was trained on large datasets including ∼333000 unlabeled sequences. Depending on the task, the model can either predict properties such as secondary structure and solubility from given protein sequence or generate amino acid sequences that match predefined structural or functional targets. Designed proteins were evaluated by computational folding and structure analysis, but they have not yet been tested in the laboratory. Later, they further advanced the design of protein sequences by proposing ForceGen, a language DM specifically designed for mechanically guided protein design [254]. Unlike MaterioFormer, which primarily focused on structure and solubility, ForceGen enabled end-to-end generation of de novo protein sequences conditioned on nonlinear mechanical unfolding responses. From a design perspective, this represents a property-driven paradigm in which target mechanical responses are specified directly rather than via predefined structural descriptors. This approach integrated a pretrained protein language model with an attention-based DM, and was trained on 7026 force-extension profiles generated from full-atom molecular simulations to learn the relationship between sequence and mechanical behavior. To generate new sequences, ForceGen used pulling force vectors as design inputs, and produced protein sequences that meet desired mechanical requirements such as toughness and strength. Another study introduced the Self-driving Autonomous Machines for Protein Landscape Exploration (SAMPLE) platform for protein engineering that combines Bayesian optimization with GP model [255]. SAMPLE employed an intelligent agent that learns from experimental sequence-function relationships to predict the activity and thermostability of proteins with different sequences and design new proteins. The agent continuously improved by interacting with an automated robotic system that tests the designed proteins and gives feedback to the model. Using this closed-loop workflow, SAMPLE designed new glycoside hydrolase enzymes with up to 12 °C improvement in thermostability. Besides designing protein sequences, Gormley and co-workers utilized active ML to design synthetic copolymers that stabilize proteins under thermal stress [256]. GPR models were built based on experimentally measured retained enzyme activity (REA) of polymer-protein hybrids (PPHs) from an initial set of 504 copolymers. Through a Learn-Design-Build-Test cycle, the model used Bayesian optimization to produce new copolymers that were then synthesized and characterized. Several copolymers were shown to effectively enhance enzyme activity after thermal treatment.

**Fig. 11:**
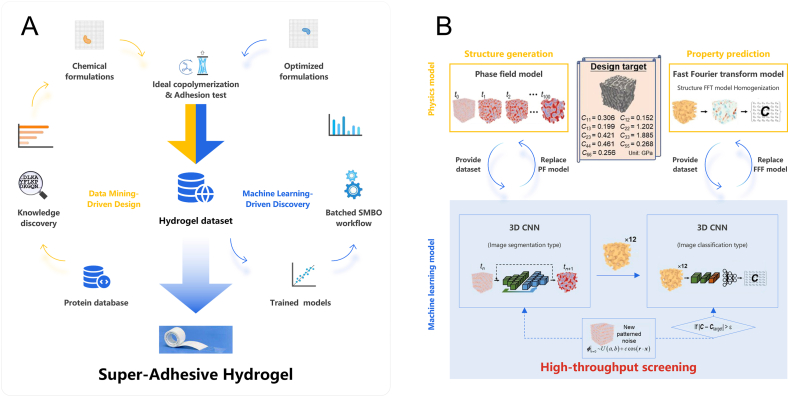
Applications of AI in the material design of bioactive materials. A) A conceptual scheme of the method combining data mining, experiments and ML to design underwater super-adhesive hydrogels [258]. B) A scheme of a CNN-based approach for rapid design and generation of spinodal structures [260].

Hydrogels are an important class of bioactive materials due to their similarity to the ECM [[306], [307], [308]], which have also been designed by AI tools. Kim and co-workers developed a ML approach to design 3D-printable hydrogel bioinks from natural materials [257]. Using ILP, they revealed a universal correlation between the rheological properties of bioinks and their printability: higher elastic modulus favors better shape fidelity and extrusion is feasible below the critical yield stress. Guided by this relationship, they applied multiple regression to construct and refine a series of bioink formulations with enhanced structure fidelity. These optimized inks were then used as supporting matrices to fabricate cell-laden hydrogel constructs, which maintained high cell viability and supported cell proliferation within the 3D environment. Gong and co-workers developed a data-driven strategy combining data mining, experimentation, and ML to design high-performance underwater adhesive hydrogels (Fig. 11A) [258]. By analyzing protein sequence patterns, they derived descriptors to guide the statistical design of 180 bioinspired hydrogels via ideal random copolymerization. Using this initial dataset, ML models, especially RFR and GP, were trained to predict the adhesive strength based on monomer compositions. The model successfully guided inverse design through SMBO, leading to the generation of novel hydrogel formulations. Several candidates were verified to exhibit exceptional adhesive strengths over 1 MPa and excellent durability. These studies reflect how AI is redefining the landscape of bioactive material design through data-driven discovery and intelligent optimization.

#### 4.1.2 Material structures

Material structures encompass features such as morphology, crystallinity, and porosity, all of which critically influence the mechanical, thermal, and functional performance of materials. Buehler and co-workers proposed a transformer-based framework that leverages natural language models to learn and abstract structural features from diatom images, facilitating the design of bioinspired architected materials [259]. A VQGAN model was trained to reconstruct the microstructure of diatoms, and integrated with Contrastive Language-Image Pretraining (CLIP) to improve the semantic-visual alignment in the generative process. The model produced synthetic 2D structural representations, which were further transformed into 3D printable designs. Selected structures were fabricated via additive manufacturing and investigated through finite element analysis to assess mechanical behavior. Banu and co-workers developed a CNN approach for rapid design and generation of spinodal structures, an architecture with stochastic but interconnected, smooth and uniform pore channels that support biological transport (Fig. 11B) [260]. Spinodal structures exhibit continuous morphologies whose local features dominate the overall structure, making them well suited for CNN. The model was built upon datasets from phase-field simulations, enabling it to replicate spinodal decomposition rapidly with significantly high computational efficiency. In parallel, a second CNN was established to predict the mechanical properties of the generated structures, allowing for inverse design based on target elasticity. On this basis, the spinodal bone structures with desired anisotropic elasticity matching natural bone were successfully created, as well as large-scale orthopedic implants with tailored gradient porosity.

Despite several studies exploring the use of AI in designing bioactive materials, most applications remain focused on property prediction. This is primarily because inverse material design requires larger training datasets and more advanced model optimization to generate materials that meet predefined property targets. Across the diverse material systems discussed above, several common patterns can be identified in AI-assisted material design. Given limited datasets and well-defined scalar outputs, most tasks employ supervised regression or probabilistic models (e.g., ANN, GP, RF) to map composition descriptors to target properties. In contrast, generative models are favored for inverse or property-conditioned design, particularly for protein sequences or complex architectures that are difficult to explore by combinatorial screening. Mechanistically, most AI models function as high-dimensional surrogates that capture implicit structure-property or sequence-function relationships. While this paradigm improves the efficiency of material development, it also introduces limitations in interpretability and data dependence. As a result, further research is needed to overcome these challenges and enable the rational design of bioactive materials through AI.

### 4.2 Fabrication optimization

The fabrication of bioactive materials entails processing materials into defined shapes and structures tailored for specific biomedical applications. Representative fabrication techniques include molding, spinning, and 3D printing, etc [[309], [310], [311]]. The quality of fabrication methods directly affects the performance of the materials. Among these techniques, 3D printing presents the high level of complexity, as it demands not only the rational design of printing models and pathways, but also the precise coordination of multiple printing parameters, such as nozzle diameters, printing speeds, and extrusion rates. In particular, bioprinting introduces additional challenges as it involves interactions between bioactive materials and living cells, and needs to maintain cell viability throughout the printing process [[312], [313], [314]]. Consequently, the successful implementation of 3D printing often requires extensive experimental optimization to adjust printing models, pathways and parameters. Moreover, even minor modifications in the material compositions may necessitate re-optimization of the entire process. AI offers a promising solution to these challenges by enabling data-driven modeling to predict the printing quality, guide the process optimization, and even monitor the fabrication process, thereby enhancing the reliability, efficiency, and reproducibility of bioactive material fabrication.

#### 4.2.1 3D printing

Fused Deposition Modeling (FDM) is an extrusion-based 3D printing technique that fabricates objects layer by layer by depositing melted thermoplastic materials through a heated nozzle. Common bioactive materials used in FDM include PLA, PLGA, and PCL, chosen for their good thermoplasticity and mechanical properties. Bayraktar and co-workers developed an ANN model to predict the tensile strength of plastic components manufactured using FDM [261]. A custom-built 3D printer, KASAME, was used to produce PLA samples under varying melt temperatures, layer thicknesses, and raster pattern orientations. Experimental results demonstrated that all three parameters had a significant impact on the mechanical strength, with the crisscross raster pattern showing the highest average tensile strength. The ANN models, trained using the Levenberg-Marquardt algorithm (LMA), accurately captured the complex relationships between processing parameters and mechanical behavior. Notably, the model associated with the crisscross pattern achieved superior predictive accuracy with the coefficient of determination (R^2^) values of 0.999199 (test) and 0.999997 (training). Koeppe and co-workers presented a neural network-based strategy for the efficient mechanical modeling of 3D-printed lattice-cell structures [262]. Lattice specimens were fabricated using PLA by FDM method, and training data were collected through mechanical testing and finite element simulations. The neural network was implemented in TensorFlow, incorporating a fully-connected layer, two LSTM cells, and a fully connected linear output layer to predict stress distributions under different loading conditions. By replacing conventional finite element simulations, this approach reduced computation time from hours to milliseconds, enabling rapid evaluation and large-scale topology optimization of complex 3D printing components. Another study used an autonomous correction system for FDM printers based on deep learning and computer vision [263]. A CNN built upon the ResNet 50 architecture was trained to classify printing quality into three categories: good-quality, under-extrusion, and over-extrusion. The ResNet 50 architecture is able to effectively capture multiscale spatial features of filament deposition. During the printing process, real-time images were continuously captured and analyzed by the trained model, which autonomously issued corrective commands via an open-source control interface to adjust the flow rate when defects were detected. This closed-loop feedback system allowed in-situ parameter optimization without human intervention, achieving over 98% classification accuracy and a correction response surpassing human reaction.

**Fig. 12:**
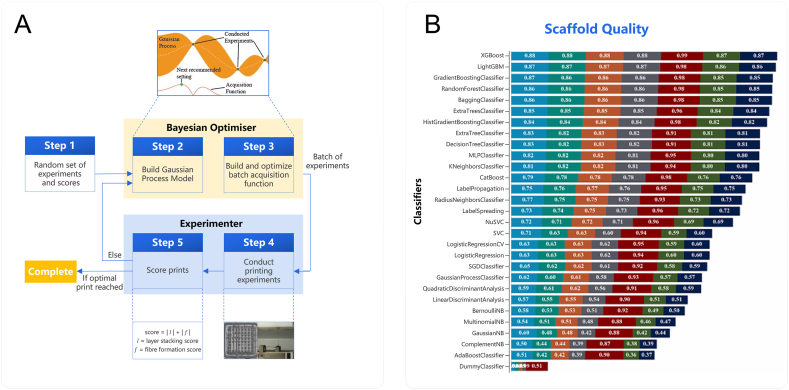
Applications of AI in the fabrication optimization of bioactive materials. A) A scheme of the Bayesian optimization model to optimize DIW printing of GelMA and HAMA bioinks [265]. B) Prediction of the scaffold quality by training algorithms [268].

Direct Ink Writing (DIW) prints structures by extruding viscoelastic inks under pressure, which are typically composed of hydrogel materials. Gu and co-workers developed a ML-based system to detect anomalies during DIW printing of GelMA hydrogels [264]. Specifically, they combined both a custom-designed four-layer CNN with a pretrained ResNeXt-50 model to classify imperfections across individual layers of the printed constructs, such as discontinuity, irregularity, and nonuniformity. Image patches extracted from layer-wise sensor data were processed and used to train the models. This system successfully identified multiple anomaly types with high accuracy reaching 90.1% and an F1-score of up to 0.955, demonstrating its potential for real-time quality monitoring and autonomous correction in 3D printing. Wallace and co-workers applied Bayesian Optimization to systematically optimize DIW printing of GelMA and HAMA bioinks (Fig. 12A) [265]. By leveraging a probabilistic model built from experimental data, the algorithm iteratively recommended optimal printer settings, including reservoir and platform temperature, extrusion pressure, and print-head speed, to improve filament continuity and structure fidelity. All experiments were conducted using an EnvisionTEC 3D Bioplotter, with quantitative printability scores serving as feedback to guide the optimization loop. This approach substantially reduced the number of experiments required, achieving high-quality lattice constructs while bypassing traditional trial-and-error processes. Kavraki and co-workers employed ML based on RF models to predict the print quality of tissue engineering scaffolds printed by DIW technique [266]. Two ML approaches were implemented: a classification model to distinguish high- and low-quality prints and a regression model to estimate quantitative quality metrics. Both models were trained on a dataset from the fabrication of poly (propylene fumarate) (PPF). The models used printing parameters (e.g., material composition, printing speed, printing pressure) as input and achieved accurate predictions of print quality.

Freeform reversible embedding of suspended hydrogels (FRESH) enables the printing of hydrogels within a support bath, preserving complex structures during fabrication, which has also been refined using ML. Washburn and co-workers presented a hierarchical machine learning (HML) framework to optimize the high-fidelity 3D printing of alginate-based biopolymers using the FRESH technique [267]. By combining physical domain knowledge with statistical modeling, they constructed an HML model incorporating rheophysical parameters (viscosity, shear rate, and pressure) derived from inputs including flow rate, printing speed, nozzle diameter, and ink concentration. Trained on a limited dataset of 48 prints, the model utilized LASSO regression to identify dominant predictors and optimize printing conditions, which is particularly advantageous for small datasets with correlated parameters. This method improved print accuracy with dimensional errors below 10%, and reduced reliance on iterative experimental testing.

#### 4.2.2 Bioprinting

Bioprinting refers to the process of printing living cells encapsulated within biomaterials, where the viability and function of the cells are influenced by printing parameters such as temperature, shear stress, and extrusion speed. Vasheghani-Farahani and co-workers applied a combination of ML and DL techniques to predict the quality of bioprinted scaffolds for tissue engineering (Fig. 12B) [268]. Leveraging an extensive dataset of 1171 samples consisting of 60 biomaterials, 49 cell lines, and different printing parameters, they trained over 40 AI models to classify cell response, printability, and scaffold quality. The study primarily focused on cell-laden bioinks composed of materials including alginate, GelMA, and hyaluronic acid, processed via DIW bioprinting. Among the applied models, five algorithms showed superior performance, which offer promising tools for the optimization of cell-laden bioprinting processes.

AI-assisted fabrication optimization commonly focuses on print quality and mechanical performance, yet different AI models have been adopted. For scalar endpoints such as tensile strength and printability scores, tabular models are commonly used to learn nonlinear mappings from process variables (temperature, speed, pressure, and layer thickness) to outcomes, offering fast screening but limited transferability across printers and materials. When the output becomes spatially distributed (e.g., stress fields) or visually defined (under/over-extrusion, layer defects), sequence models are preferred due to their ability to represent field evolution or multiscale filament morphologies. Although AI has been widely applied to optimize 3D printing processes, its use in other fabrication methods remains relatively limited. Moreover, AI-driven research in bioprinting is even less explored due to the added complexity of embedded living cells. Therefore, substantial research is still required to advance AI integration in the bioprinting.

### 4.3 Property prediction

In the conventional development of bioactive materials, a series of property tests are often required to determine whether materials meet the application standards. This process often involves numerous parallel experiments and relies on advanced analytical instruments, making it time-consuming and resource-intensive. With the integration of AI, these properties can be accurately predicted based on chemical compositions and fabrication parameters, thereby reducing experimental workload and accelerating development processes. This section highlights the use of AI in predicting glass transition temperature (T_g_), mechanical properties, solubility, degradability, nanozyme activity, hydrogel formation, protein characteristics, and protein adsorption.

#### 4.3.1 T_g_

T_g_ is the temperature at which a polymer transitions from a rigid, glassy state to a flexible, rubbery state, reflecting the mobility of polymer chains. This study developed a ML framework to predict the T_g_ of polyhydroxyalkanoate (PHA) homopolymers and copolymers [269]. They used a curated dataset of 133 polymers and applied RF models trained on molecular descriptors derived from topology, shape, and polarity. The model not only achieved high predictive accuracy for T_g_ but also revealed chemical trends, providing a meaningful way to design PHA compositions with desired thermal properties. Washburn and co-workers employed ML to predict the T_g_ of linear polyurethane elastomers [270]. A dataset comprising 43 distinct thermoplastic polyurethanes (TPUs) was constructed and 40 descriptors were extracted from quantum mechanical, cheminformatics, and solubility calculations. RF models were trained and refined through permutation importance-based features and collinearity analysis. The resulting sparse model successfully predicted T_g_ and identified key structural determinants underlying the thermal behavior of these materials.

#### 4.3.2 Mechanical properties

**Fig. 13:**
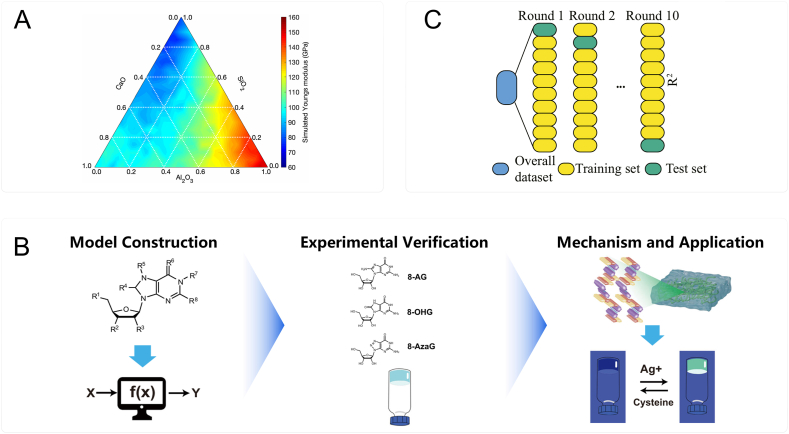
Applications of AI in the property prediction of bioactive materials. A) A ternary diagram illustrating the Young's modulus of calcium aluminosilicate glasses predicted by high-throughput molecular dynamics simulations [271]. B) A scheme of using ML to predict whether nucleoside derivatives can form supramolecular hydrogels [278]. C) A scheme of the tenfold cross-validation to evaluate the predictive accuracy of the model [284].

Mechanical properties typically include tensile strength, elasticity, modulus, toughness, and elongation at break, etc. The Young's modulus of calcium aluminosilicate (CAS) glasses has been predicted by ANN and high-throughput molecular dynamics simulations (Fig. 13A) [271]. A comprehensive dataset composed of 231 simulated compositions was constructed to span the ternary CAS system. The MLP model exhibited high prediction accuracy (R^2^ = 0.975) and effectively captured the non-linear compositional relationship between compositions and mechanical stiffness. Javaid and co-workers utilized ML to systematically identify and rank suitable biomaterials for bone scaffold applications [272]. They established a dataset comprising 17 candidate materials and extracted three critical mechanical properties including compressive strength, tensile strength, and Young's modulus, which were further augmented through simulations. Six supervised ML algorithms were evaluated and ANN showed the highest classification accuracy of 96.5%. Subsequently, Preference Ranking Organization METhod for the Enrichment of Evaluations (PROMETHEE) was applied to rank the candidate materials, identifying Brushite and Titanium alloys as the most appropriate materials for cancellous and cortical bone scaffolds, respectively.

#### 4.3.3 Solubility

Solubility is the ability of bioactive materials to dissolve in solvents to form homogeneous solutions. This study leveraged an ensemble ML approach to forecast the dissolution behavior of biomedical oxide glasses [273]. A hybrid model combining RF with AR was trained on a dataset of 1364 records spanning five compositional systems and diverse physiochemical parameters (e.g., pH, temperature, surface area). By modeling time- and composition-dependent ion release profiles, the model successfully captured Stage I and II dissolution kinetics across a wide pH spectrum. Compared to six standalone algorithms, the ensemble model demonstrated superior predictive performance and allowed rational design of glasses with controlled solubility.

#### 4.3.4 Degradability

Degradability is an important property for bioactive materials, especially those intended for implantation, as it affects their interaction with biological environments over time. ML was utilized to quantify the biodegradation of type I collagen scaffolds induced by osteogenic differentiation of hMSCs [274]. A key technical innovation lies in the use of a supervised RF classifier to perform image segmentation based on physically interpretable texture descriptors, specifically structure tensor and entropy, enabling quantitative analysis of collagen degradation from optical microscopy images. From the segmented regions, features associated with cellular growth and collagen degradation were extracted and modeled over time using both parametric and nonparametric regression approaches, including logistic mixture models. This study exhibited accurate estimation of scaffold degradation, elucidating the relationship between cellular activity and collagen mass loss.

Nazarpak and co-workers employed AI to predict the degradation rate of genipin-crosslinked gelatin scaffolds [275]. Two supervised learning algorithms, ANN and KRR, were trained using experimentally derived features including gelatin/genipin ratio, swelling behavior, pore size, mechanical strength, and the degree of crosslinking. Degradation rates measured at five time points were utilized for model assessment. The ANN model achieved superior performance with a mean squared error (MSE) of 2.68%, underscoring its potential for guiding scaffold design in tissue engineering.

#### 4.3.5 Nanozyme activity

Nanozymes are a class of nanomaterials that exhibit enzyme-like catalytic activities, exhibiting enhanced stability and controllability compared to natural enzymes. This study applied the DNN model to predict and design nanozymes [276]. They used a selected dataset of 920 entries derived from literature and trained the models based on internal and external factors. The models achieved high predictive accuracy of 90.6% and R^2^ values up to 0.80, allowing both type identification and activity quantification of nanozymes. Sensitivity analysis further highlighted the dominant role of transition metals in determining nanozyme activity, offering a rational framework for nanozyme design.

#### 4.3.6 Hydrogel formation

Hydrogel formation is the process in which 3D polymer networks are formed in water by physical or chemical crosslinking. This study employed ML to predict the gelation behavior of 2304 peptidelike compounds synthesized via the Ugi four-component reaction [277]. Molecular descriptors of over 7 million structural features were generated using PaDEL. To address class imbalance, three common resampling methods were applied. After data resampling, multiple classification algorithms were tested and GB showed the highest precision. This superiority arises because GB incrementally focuses on hard-to-classify minority samples, which is critical given the extremely imbalanced gelation dataset. The predicted hydrogels exhibited tunable stiffness related to nanofiber structures and supported cell proliferation, confirming their biocompatibility and potential for biomedical applications. Zhao and co-workers developed a ML model to forecast whether nucleoside derivatives can form supramolecular hydrogels (Fig. 13B) [278]. Based on 71 reported compounds, the optimal LR model achieved an accuracy of 71%. Following experimental verification of 24 predicted candidates, several new gelators were identified, including two rare cation-independent hydrogels with potential applications in visual detection of Ag^+^ and cysteine.

#### 4.3.7 Protein characteristics

Proteins are a class of biological macromolecules with complex molecular compositions and secondary structures, which decide their properties such as solubility, activity, natural frequency, and specific binding interactions. Buehler and co-workers leveraged end-to-end DL to predict the secondary structure content of proteins directly from their amino acid sequences [279], focusing on global structural descriptors rather than residue-resolved assignments. Trained on over 120000 proteins from the Protein Data Bank (PDB), the model effectively captured sequence-structure relationships and accurately predicted alpha-helix and beta-sheet contents. Furthermore, it also enabled de novo design of protein sequences with customized structural features, which was verified by AlphaFold2. In addition, the authors also presented a GNN framework to predict protein natural frequencies from amino acid sequences and low-resolution structural inputs, such as contact or distance maps [280]. Using a dataset of over 34000 proteins and employing PNA, the model achieved end-to-end prediction of frequency spectrum while reducing computation time compared to normal mode analysis, particularly in the absence of high-resolution structures.

#### 4.3.8 Protein adsorption

Protein adsorption is the process where proteins adhere to the surface of materials, which influences subsequent biological responses. The protein adsorption and cellular response to the surfaces of biodegradable polyarylates were explored using ANN [281]. Over 800 molecular descriptors were produced by Molecular Operating Environment (MOE) and Dragon software, and key features were selected through Monte Carlo (MC) analysis. The resulting ANN models achieved reliable predictions using only five inputs, enabling efficient biomaterial pre-screening. Yarovsky and co-workers employed ML to quantify protein adsorption on functionalized self-assembled monolayers (SAMs) by using lysozyme and fibrinogen as representative contaminants [282]. Two QSPR models were trained on 176 SAM-protein interaction data with 67 molecular descriptors. The optimized models (R^2^ = 0.82) elucidated key features that affect antifouling performance. Later, Hayashi and co-workers applied ANN to quantitatively predict the water contact angle and protein adsorption on SAMs from molecular structures [283]. Built upon 145 experimental SAMs, the model captured key structural descriptors and achieved accurate predictions. It was further extended to evaluate hypothetical SAMs, demonstrating its promise for material screening and biointerface design. Another study used ML to forecast the functional composition of the protein corona and cellular recognition of nanoparticles (Fig. 13C) [284]. A RF model was trained with 652 data points collected through meta-analysis, incorporating 21 physicochemical and experimental factors. This model showed high accuracy with R^2^ over 0.8 in predicting corona components such as immune proteins, apolipoproteins, and complement proteins, and successfully anticipated macrophage uptake and cytokine release.

Based on AI, different properties of bioactive materials can now be accurately predicted. However, most studies prioritize predictive performance over systematic model benchmarking, making it difficult to identify the respective contributions of model selection, feature representation, and data quality to observed performance improvements. Moreover, mechanistic interpretation is not adequately addressed, with AI models primarily serving as data-driven surrogates rather than explicitly linking learned patterns to underlying physicochemical mechanisms. In addition, current models remain highly specific, which requires retraining for each distinct material or target property. This underscores the need for a universal model that can adapt across different material classes and property types to accelerate the design and prediction of bioactive materials.

### 4.4 In vitro assessments

In vitro assessments refer to the systematic evaluation of the interactions between bioactive materials and cells in vitro. These assessments encompass a wide range of biological processes, including cell adhesion and differentiation, as well as cytotoxicity, metabolism, antimicrobial activity, and immune responses. In vitro assessments are essential for preliminary validation of material biocompatibility and functionality, which are a prerequisite for subsequent in vivo studies. The integration of AI can predict the interactions between bioactive materials and cells, thereby potentially enhancing the efficiency and scalability of in vitro assessments.

#### 4.4.1 Cytotoxicity

**Fig. 14:**
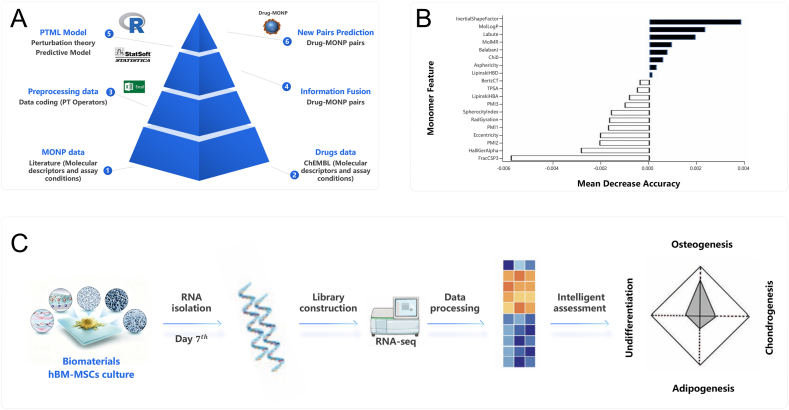
Applications of AI in the in vitro assessments of bioactive materials. A) A scheme of predicting the DDNS toxicity by the PTML model [288]. B) Mean decrease accuracy of descriptors calculated by summing the importance of all monomer features [292]. C) A scheme of a MeD-P framework to predict the biomaterial-induced lineage fate of hMSCs [295].

Cytotoxicity is the degree to which materials can kill living cells. Leszczynski and co-workers established a nano-QSAR model to predict the cytotoxicity of 18 metal oxide nanoparticles to HaCaT cells [285]. By combining experimental toxicity testing, quantum-mechanical descriptors and image descriptors, the model revealed dual mechanisms of toxicity, achieving accurate in silico prediction and mechanistic interpretation. Later, Pan and co-workers developed nano-QSAR models using SMILES based descriptors integrated with basic physicochemical features, eliminating the need for quantum-mechanical and image descriptors [286]. Applied to HaCaT cells, the model demonstrated high predictive accuracy while offering enhanced accessibility, computational efficiency, and mechanistic insight, thus simplifying cytotoxicity prediction for metal oxide nanoparticles. Another study proposed a quasi-QSAR model to forecast the viability of human bronchial epithelial cells (BEAS-2B) and HaCaT cells exposed to 21 metal oxide nanomaterials based on a comprehensive toxicity dataset [287]. By encoding experimental conditions and physicochemical properties into quasi-SMILES using hierarchical cluster analysis (HCA) and modeling with CORAL software, this approach significantly improved the accuracy and robustness of cytotoxicity prediction. The biological activity and toxicity were also predicted by perturbation-theory machine learning (PTML) models that integrate perturbation operators from over 500000 nanoparticle drug delivery systems (DDNS) (Fig. 14A) [288]. The selection of PTML can be attributed to its ability to capture condition-dependent variability in highly heterogeneous nanoparticle-biological datasets. The models were trained on preclinical assay data under different experimental conditions and exhibited 94.5% accuracy, supporting the selection of suitable drugs, coating agents, and metal oxide nanoparticles for DDNS design.

#### 4.4.2 Cell adhesion

Cell adhesion to bioactive materials is mediated by the interactions between the cell membranes and the material surfaces. Davies and co-workers employed high throughput surface characterization to investigate the influence of acrylate polymer surface chemistry on human embryoid body (hEB) cell adhesion [289]. Using PLS regression, they analyzed 496 polymer spots to identify surface moieties associated with protein adsorption and cellular response, revealing specific chemical features that either promote or inhibit adhesion. Building on this work, the authors developed purely computational models using BNN trained on molecular descriptors derived from monomer structures [290]. Without relying on experimental surface data, this approach accurately predicted the adhesion of hEB cells, demonstrating that cell adhesion can be reliably inferred from polymer components. Kalyon and co-workers established a ML framework to quantify and classify single-cell confinement in melt electrowritten PCL scaffolds with tunable 3D architectures [291]. Based on morphological and cellular features from fibroblasts, the SVM classifier achieved high accuracy in distinguishing shape phenotypes, revealing that woven substrates induced superior morphological homogeneity. In addition, hydrogel coatings capable of resisting platelet adhesion on implantable biosensors were identified with a high-throughput platform (Fig. 14B) [292]. RF models built upon molecular descriptors of polyacrylamide copolymers enabled the discovery of nonintuitive formulations that surpassed PEG in anti-biofouling performance, thereby enhancing in vivo sensor performance and allowing sustained drug monitoring under biofouling-prone conditions.

#### 4.4.3 Cell differentiation

Cell differentiation is the biological process where unspecialized cells develop into more specialized cell types with specific functions. This study established a DL-based model for predicting the differentiation of neural stem cells (NSCs) using brightfield single-cell images [293]. An Xception-based CNN model was trained on the images of NSCs exposed to diverse inducers, including hormones, neurotrophins and nanoparticles. This approach achieved the identification of cell differentiation at early stages, demonstrating high generalizability and robustness. To understand and optimize the differentiation of NSCs on engineered interfaces, Lee and co-workers developed a high-throughput platform using laminin-coated, glass-based combinatorial nanoarrays fabricated via dynamic laser interference lithography (DLIL) [294]. Based on GPR models, this study enabled cell-type-specific prediction of the differentiation in NSCs derived from adult and human-induced pluripotent stem cells (hiPSCs) across diverse nanotopographies.

Besides NSCs, the differentiation of hMSCs was also explored using AI. Deng and co-workers reported a MSCs Differentiation Prediction (MeD-P) framework to predict biomaterial-induced lineage fate of hMSCs (Fig. 14C) [295]. By employing a kNN algorithm trained upon RNA-seq datasets spanning osteogenesis, chondrogenesis, and adipogenesis, MeD-P achieved high accuracy of 90.63% and early-stage differentiation assessments across different biomaterials. Another study developed a DL approach to predict the osteogenic differentiation of rat bone marrow mesenchymal stem cells (rBMSCs) cultured on various biomaterial surfaces, including titanium with different nanotopographies and coatings such as collagen I, hyaluronic acid, amyloid fibrils, and polydopamine [296]. An osteogenic CNN (OCNN) was trained using laser scanning confocal microscope images of single cells stained with F-actin and DAPI. The model demonstrated high predictive accuracy at early stages of osteogenic differentiation and outperformed SVM. OCNN can be further optimized through online learning to accommodate different material types.

#### 4.4.4 Metabolism

Metabolism of bioactive materials refers to their biochemical degradation, transformation, or assimilation within biological systems. Chan and co-workers developed a supervised DNN to forecast the in vivo fate of PEGylated AuNPs [297]. By integrating dynamic proteomic features with quantitative biodistribution profiles, the model achieved up to 94% accuracy in predicting hepatic and splenic accumulation. Notably, by employing the host as a bioreactor, nanoparticles with tunable surface coronas were engineered, resulting in controlled in vivo degradation and reducing liver and spleen uptake by 50% and 70%, respectively.

#### 4.4.5 Antimicrobial activity

Antimicrobial activity is the ability of bioactive materials to kill or inhibit microorganisms such as bacteria, fungi, or viruses. This study applied neurofuzzy logic technology to analyze a literature-derived dataset linking the compositions and processing conditions of BG to antibacterial performance [236]. The model clarified variability across different studies and identified key parameters that determine the antimicrobial activity. These results offered interpretable, data-driven guidance for the rational design of antibacterial BG and support their applications in targeting common pathogens associated with skin and implant-related infections.

#### 4.4.6 Immune responses

Immune responses denote the biological process where the body recognizes and reacts to foreign substances. Alexander and co-workers utilized high-throughput screening on polystyrene-based TopoChips to examine the influence of microscale surface topographies on human monocyte attachment and macrophage differentiation [298]. A library of 2176 algorithm-generated patterns were tested, identifying that micropillars with diameters of 5-10 μm were dominant determinants of cell attachment. GB regression was applied to correlate attachment responses with 65 topographical descriptors, revealing that both pillar size and density play a key role in modulating macrophage polarization. This study established clear structure-function relationships between surface design and immune response, providing design principles for creating anti-inflammatory biomaterial surfaces. The effect of nanotopographies on macrophage polarization was also studied using DLIL technology [299]. The authors fabricated a combinatorial biophysical cue (CBC) array containing over one million nanotopographies on glass, silicon, and clinically relevant titanium substrates. GPR algorithm was employed to predict topographical patterns that drive pro-inflammatory (M1) or anti-inflammatory (M2) responses, since it enables continuous mapping and interpolation of sparse, spatially distributed cellular responses. The predictions were experimentally verified, revealing principal mechanisms such as cytoskeletal remodeling and Rho-associated protein kinase (ROCK) dependent epigenetic activation. This integrated platform enabled precise nanostructure design for immune modulation and enhanced the performance of biomedical implants.

Ghaemmaghami and co-workers used high-throughput polymer microarray screening to identify simple (meth)acrylate and (meth)acrylamide polymers capable of directing macrophage polarization towards M1 or M2 phenotypes [300]. The immunomodulatory functions of selected candidates were tested in a murine foreign body model, showing in vivo immune responses consistent with in vitro findings. To illustrate chemical features that decided immune-instructive behavior, three ML models (RF, SVM, MLP) were constructed to predict macrophage responses based on polymer chemical descriptors. These results opened new avenues for the rational development of immune-instructive polymers with broad biomedical applications. Another study predicted the immunomodulatory properties of anti-inflammatory polymers using ML [301]. A dataset was generated from cellular assays conducted on 50 distinct polymers using RAW 264.7 murine macrophages. A BLR model was developed using this dataset. Subsequently, two models-KNN and Naïve Bayes-were implemented to forecast anti-inflammatory properties. The results indicated that polycationic polymers were three times more likely to exhibit anti-inflammatory effects, and nitric oxide secretion served as a reliable indicator for determining the anti-inflammatory ability. This study demonstrated that ML can accelerate the discovery of immunomodulatory biomaterials with limited experimental data.

In the above studies, AI helps interpret material-immune interactions by learning mappings between material descriptors and immune readouts. By quantifying the relative contributions of coupled material parameters, ML models identify dominant immunomodulatory cues and provide insight into how material properties relate to intermediate cellular processes, such as cytoskeletal remodeling and mechanotransduction.

For in vitro assessments, AI models are typically trained to predict experimental readouts such as cell viability, marker expression, or morphology-based scores. Although these endpoints are convenient for high-throughput analysis, they represent phenotypic outcomes instead of underlying mechanisms. As a result, predictive accuracy on assay measurements is emphasized, whereas mechanistic interpretation of material-cell interactions remains limited. Additionally, the intrinsic gap between in vitro models and the highly dynamic in vivo environments restricts their translational relevance. Further in vivo investigations remain essential to elucidate material-tissue interactions under physiological conditions and provide high-quality data for AI-assisted in vivo assessments.

### 4.5 In vivo assessments

In vivo assessments denote the evaluation of bioactive materials in living systems, which is essential for their biomedical applications and clinical translation. Given the complexity of biological systems, in vivo assessments are often intricate and challenging. With the aid of AI, the performance of bioactive materials in vivo can be potentially predicted and underlying mechanisms can be further clarified.

**Fig. 15:**
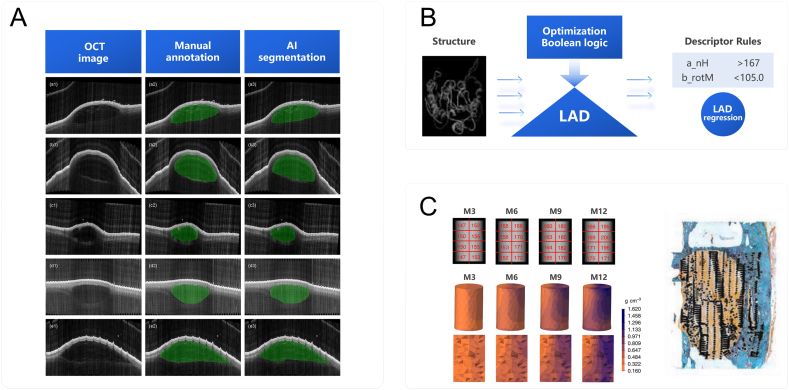
Applications of AI in the in vivo assessments of bioactive materials. A) Representative cross-sectional OCT images with manual annotation and AI segmentation [302]. B) Schematic overview of the LAD-based modeling approach used to correlate polymer structures with DNA transfection performance [303]. C) Comparison of AI-based in silico predictions and in vivo histological results for scaffold-supported bone regeneration in a sheep tibial defect [304].

Werkmeister and co-workers quantified the intradermal volume of hydrogels based on DL [302]. A custom-built optical coherence tomography (OCT) system was employed to acquire volumetric data of mouse skin injected with soft-tissue fillers (Fig. 15A). To analyze these complex biomedical images, a CNN model with a modified U-net architecture was trained on 2D slices from 100 manually annotated 3D datasets. This DL approach achieved high accuracy (0.9938) and Jaccard similarity (0.879), supporting reliable quantification of filler volume and degradation in vivo.

In addition, the structure-activity relationships in polymeric gene delivery were investigated by applying LAD to a library of poly (β-amino esters) [303]. Linear combination and explicit representation models were introduced to correlate polymer structures with DNA transfection efficiency (Fig. 15B). Among various regression approaches, LAD delivered the highest predictive accuracy with Pearson correlation coefficient of 0.77, owing to its robustness against noisy and limited datasets and its ability to handle classification errors and missing attributes that degraded the performance of other regression models. This study provided a computational framework to support rational design of biodegradable polymeric gene vectors.

The bone regeneration process within porous tissue scaffolds was also studied using a ML-based multiscale model [304]. The model employed three neural networks to learn the mechanical responses and remodeling behavior from a 12-month in vivo study on the repair of a major segmental defect in sheep tibia with ceramic scaffolds (Fig. 15C). Compared to the multilevel finite element model, this approach reduced computation time without compromising predictive accuracy, enabling efficient and personalized simulation of long-term bone ingrowth for scaffold-based repair of critical bone defects.

AI offers a promising approach to facilitate in vivo assessments of bioactive materials. Yet, existing research is still limited and predominantly relies on simple models and short-term evaluations. Considering the complex and long-term nature of in vivo processes, further in-depth studies are required to enable more comprehensive biological evaluation and accelerate translation toward clinical applications.

### 4.6 Summary of AI applications

Taken together, this section highlights a process-oriented framework and utilizes numerous case studies to illustrate the diverse applications of AI in bioactive materials. The discussion spans material design, fabrication optimization, property prediction, in vitro assessments, and in vivo assessments. Considering the inherent complexities associated with bioactive materials, some of these topics are still in the nascent stages of research. For instance, AI-driven materials design, bioprinting, and in vivo studies are underexplored topics and require further research. Furthermore, the ultimate clinical translation of the AI-based innovations faces significant hurdles in terms of data standardization, model interpretability, and evolving regulatory frameworks, which will be discussed in the following section.

## 5 Challenges and opportunities in bioactive materials

### 5.1 Regulatory hurdles

The integration of AI into bioactive materials development presents unprecedented opportunities alongside formidable regulatory and technical challenges. These dual aspects are intrinsically interwoven, collectively shaping the trajectory of this rapidly evolving field. A pivotal challenge lies in establishing a scientific, efficient, and adaptive regulatory framework capable of keeping pace with accelerated technological innovation. While the U.S. Food and Drug Administration (FDA) serves as a critical global benchmark, a comprehensive analysis must encompass the diverse regulatory landscapes of other major jurisdictions, including Europe and Asia, to reflect the truly global nature of this endeavor.

As of mid-2025, the FDA has not yet issued standalone, comprehensive regulations specifically governing AI-assisted bioactive materials or their combination products. This regulatory uncertainty itself constitutes a significant challenge for current research and development activities.

Nevertheless, analysis of FDA's evolving posture since 2023 reveals a proactive, multi-layered strategy. Firstly, the agency has adopted an approach centered on issuing discussion papers and soliciting stakeholder feedback, such as the 2023 Discussion Paper on Using AI and ML in the Development of Drug and Biological Products [315]. Secondly, the FDA is actively exploring the translation of regulatory experiences gained from “Software as a Medical Device” (SaMD) to broader AI applications in biomaterials. The conceptual framework of the Predetermined Change Control Plan (PCCP) for managing iterative updates of AI models holds significant relevance for material design models requiring continual refinement [316].

In Europe, the regulatory environment for AI in healthcare is shaped significantly by the Medical Device Regulation (MDR) and the active guidance of the European Medicines Agency (EMA). The MDR provides the core framework for devices incorporating AI, emphasizing a risk-based classification and requiring robust clinical evaluation and post-market surveillance. For AI-driven bioactive materials that constitute a drug-device combination product, the evaluation follows a similar principle of identifying the principal mode of action, involving either a notified body (for device-led) or a national competent authority/EMA (for drug-led) [209]. The EMA has been proactive in considering the use of AI in medicinal product development. Its 2024 reflection paper on AI in the medicinal product lifecycle outlines key principles for data quality, algorithm transparency, and robust validation, stressing that existing regulatory standards for quality, safety, and efficacy remain fully applicable [317]. A distinctive aspect of the EU framework is the proposed Artificial Intelligence Act, which aims to establish a horizontal regulatory framework for AI across all sectors. When enacted, its provisions for high-risk AI systems, including certain medical applications, will impose additional requirements concerning risk management, data governance, technical documentation, and human oversight, which will intersect with existing medical product regulations [318]. Following its departure from the European Union, the United Kingdom operates an independent regulatory framework overseen by the Medicines and Healthcare products Regulatory Agency (MHRA). Rather than adopting a horizontal legislative framework such as the EU Artificial Intelligence Act, the UK follows a sector-specific regulatory strategy in which existing regulators oversee AI within their areas of responsibility. In healthcare, the MHRA has initiated the Software and AI as a Medical Device (SaMD/AIaMD) Change Programme to develop tailored requirements that protect patient safety, address algorithmic bias, and support lifecycle oversight of adaptive AI systems while maintaining a pro-innovation regulatory environment.

Major Asian regulatory agencies are also developing tailored strategies. China's National Medical Products Administration (NMPA) has released guidelines for AI medical device software, emphasizing a lifecycle regulatory approach and the necessity of providing “de-black box” evidence, methods to interpret and understand AI decision-making processes, which directly impacts the validation of AI models for material design [319]. Japan's Pharmaceuticals and Medical Devices Agency (PMDA) promotes a “regulatory science” approach, offering early consultation schemes (“Sakigake”) for innovative technologies. The PMDA acknowledges the potential of in silico methods and AI, encouraging dialogue on virtual trial designs and model validation strategies for advanced therapies and materials [320]. Similarly, South Korea's Ministry of Food and Drug Safety (MFDS) has updated its guidelines to incorporate digital healthcare technologies, including AI, and is establishing new review pathways for AI-based medical products, focusing on performance evaluation based on real-world data and continuous learning management [321].

Across these regions, common technical validation hurdles persist. Regulators universally grapple with establishing trust in AI model predictions for complex biological responses like biocompatibility. The core demand is for “fit-for-purpose” validation using a risk-based approach, requiring robust evidence on data representativeness, rigorous performance metrics, and mitigation of bias and opacity [310,311]. While specific standards for AI-generated biocompatibility data (e.g., complementing ISO 10993) are still emerging, comprehensive documentation detailing algorithm design, data provenance, and lifecycle management is becoming a global expectation. Initiatives like the International Medical Device Regulators Forum (IMDRF) are working towards harmonizing definitions and principles for SaMD and AI as a Medical Device, which will provide a foundational layer for global regulatory convergence [312].

Beyond regulatory adaptation, significant opportunities lie in overcoming these challenges. Addressing data scarcity is driving innovations in automated high-throughput experimentation and characterization platforms, specifically designed to generate the rich, standardized datasets AI thrives on. Concurrently, the field of Explainable AI (XAI) is rapidly advancing, developing methods to elucidate the reasoning behind complex model predictions, enhancing scientific trust, revealing hidden material-biology correlations, and directly supporting regulatory review. Furthermore, the integration of multi-scale computational modeling (quantum mechanics, molecular dynamics, finite element analysis) with AI forms a powerful synergy. AI can parameterize and accelerate these physics-based models, while mechanistic models provide foundational understanding that can constrain and guide AI learning, leading to more interpretable and physically plausible predictions of bioactive material behavior across scales.

In conclusion, the path forward for AI in bioactive materials necessitates a concerted global effort. Overcoming data limitations, enhancing model transparency, and establishing robust validation protocols are critical scientific challenges. Simultaneously, proactive and harmonized evolution of regulatory science evident in the flexible learning-based approaches being explored by the FDA, EMA, MHRA, NMPA, PMDA, and MFDS, is essential to translate AI's potential into clinically impactful and safely regulated bioactive material innovations worldwide.

### 5.2 Ethical issues related to AI in bioactive materials science and engineering

AI in bioactive materials research, like many other AI-assisted domains, is not free from ethical challenges. These concerns arise from both the rapid advancement of AI technologies and the sensitive nature of bioactive materials science, which operates at the intersection of materials engineering and medical science. This section highlights the ethical issues most relevant to the field. Issues related to intellectual property and interpretability are discussed elsewhere, as they are common to most AI models regardless of the domain of application.

Data-related ethical issues constitute an especially significant category of concerns-particularly for models trained on clinical outcomes, including medical imaging, free-text data and genetic information. These concerns often stem from inadequate anonymization procedures and the risk of re-identification of sensitive medical data used during model training. It seems that Federated Learning (FL) could be used as remedy for the mentioned issues. This AI paradigm assumes distributed processing of sensitive data instead of doing this centrally. Since data is not shared with the central server, but scattered across a large number of clients, the risk of patient data leakage is reduced to some extent [322]. A related issue involves determining ownership and control of AI models developed using medical datasets. Additional data-related concerns include bias and representativeness [323]. When AI systems are trained on data derived from limited populations or specific ethnic or age groups, their predictions may not generalize well to broader patient populations. This can result in unequal performance of AI-designed bioactive materials across demographic groups and potentially harmful outcomes for underrepresented populations. Bias can be effectively reduced by using diverse and representative datasets; however, learning approaches that explicitly focus on underrepresented groups in existing data - such as active learning - are also considered beneficial [324]. Furthermore, generative AI systems may fabricate data appearing credible but incorrect-a phenomenon commonly referred to as AI hallucination [325]. If not carefully verified, such outputs may threaten the reliability and reproducibility of research findings.

Safety and translational risks constitute another major ethical concern. One key issue involves accountability: if AI-assisted design leads to the failure of bioactive material after implantation or administration, it remains unclear whether liability lies with the developer of the AI system, the materials scientist, the clinician, or the regulatory body. There is also the risk of misusing AI-assisted material design to optimize harmful bioactive compounds, develop toxic delivery systems, or create materials intended for non-therapeutic biological manipulation.

It is well recognized that AI has the potential to reduce animal experimentation, which is ethically desirable. However, this benefit depends upon the reliability and proper validation of AI models. Overreliance on insufficiently validated systems may result in inadequate biological testing or misleading assumptions regarding safety and efficacy. Additionally, the possibility of using AI-designed bioactive materials to manipulate biological systems raises further ethical concerns, particularly in the absence of clear regulatory frameworks.

Finally, there is an epistemological dimension to consider. Excessive reliance on AI may lead to the under exploration of scientific creativity. Researchers may become increasingly focused on parameter optimization and performance metrics at the expense of mechanistic hypothesis development and deep conceptual understanding.

### 5.3 Application limitations and future directions

AI playes an important role across different development stages of bioactive materials, ranging from material design to biological assessments. Nevertheless, the intrinsic complexity of bioactive materials still imposes significant limitations on AI-assisted material development. One major challenge is that current research predominantly employs AI for property prediction, while its potential for material design has yet to be fully explored. Achieving material design demands not only extensive and reliable datasets to train models, but also iterative refinement of algorithms to extract hidden patterns and underlying relationships. The identification of these principles is a prerequisite for using AI in the inverse design of materials with desired properties. Although this process is time- and resource-intensive, it remains worthwhile as the rational material design can minimize subsequent trial-and-error and facilitate practical applications.

Another limitation lies in fabrication optimization. To date, AI-driven fabrication optimization has been applied primarily to 3D printing owing to its significantly greater complexity. However, research on 3D bioprinting is still scarce because it requires simultaneous control of material properties and cell behaviors to ensure the cell viability during the printing process, which is more challenging than conventional 3D printing. Even so, 3D bioprinting represents a crucial future direction as it allows the direct co-fabrication of cells and biomaterials into predefined architectures, providing a foundation for organ fabrication [326]. With the emergence of new 3D bioprinting approaches [327,328], the demand for AI to enhance precision, efficiency, and reproducibility in this field is becoming increasingly urgent.

Finally, most AI applications in bioactive materials are confined to material and cell levels. Yet, the ultimate validation requires systematic in vivo testing, where the physiological environment differs markedly from in vitro conditions. Immune responses, degradation dynamics, and tissue regeneration further complicate the in vivo evaluation, making the assistance of AI in this process both crucial and challenging. One promising solution is to integrate AI with complementary approaches such as digital twin [329], multi-scale modeling [330], computational biomechanics [331], etc. By combining these approaches, the biological complexity could be captured more realistically. Such strategies hold potential to bridge the gap between in vitro prediction and in vivo performance, thereby advancing the development and clinical translation of bioactive materials.

Conversely, synthetic biology constitutes a domain in which AI methodologies are anticipated to be especially powerful and transformative, given the field's complexity, high-dimensional data structures, and design-oriented nature. Synthetic biology has emerged as a powerful scientific discipline with significant potential to promote the biology-driven transformation of materials science, particularly in the field of bioactive materials [332]. In this context, the term Materials Synthetic Biology (MSB) was coined and defined as the “integration of engineering principles from synthetic biology and materials science to redesign living systems as dynamic and responsive materials with programmable and emergent functionalities”.

Within synthetic biology, cells can be engineered to produce specific building blocks for the chemical synthesis of diverse materials. Another important approach involves engineered living materials (ELMs). ELMs have been defined as engineered materials composed of living cells that either form or assemble the material itself or modulate its functional performance in a controlled manner. In the case of ELMs, cells are deliberately programmed to generate structured, functional living materials. Among numerous examples, one can mention living electronic devices, self-regulated drug depots, and bacteria-based ELMs designed to produce therapeutic compounds in response to optical stimuli [[333], [334], [335]], in which the cells remain an integral and active component of the system.

The application of AI in MSB could be highly beneficial. Initially, ML algorithms were primarily used to predict protein structures from amino acid sequences. However, with the development of deep learning models based on transformer architectures, AI systems can now perform far more complex tasks, including predicting functional and physical outcomes directly from nucleic acid sequences [336].

An example of these expanded capabilities is the work of Qu and co-workers, in which a large language model (LLM)-based approach was presented to automate and enhance gene-editing experiments [337]. The proposed model demonstrated the potential to improve the efficiency and precision of DNA editing using Clustered Regularly Interspaced Short Palindromic Repeats (CRISPR) technology.

Future generative AI systems are expected to move beyond simple prediction and classification tasks, potentially evolving into intelligent biological design platforms capable of understanding and adapting to the complex contextual challenges associated with engineered living materials. Advancing this intersection of AI and synthetic biology responsibly will require proactive governance grounded in continuous knowledge development, accountability, transparency, and strong ethical principles.

### 5.4 Data standardization challenges

The use of AI methods in the design of new bioactive materials will certainly intensify in the future. However, for this to happen, we should set the right directions for action and identify measures that would enable more efficient ways of using AI methods in materials engineering. One of the desired directions of development is the universalization of ways of representing materials in AI models. New ways of representing materials should enable the most comprehensive recording of the chemical and physical properties of different classes of materials (polymers, metallic materials, ceramics). It can be assumed that feature extraction based on deep learning (e.g., using GNNs) may play a special role in automating the discovery of such universal descriptors [338].

An important obstacle to the application of AI in bioactive materials engineering is the lack of standardization in the data used to train AI models. Datasets are often heterogeneous, lacking uniform formats, consistent metadata structures, and standardized terminology, which makes it difficult to integrate data from different sources. To overcome this challenge, it is essential to ensure semantic interoperability, i.e., the ability of datasets to be exchanged and interpreted with a shared understanding of their meaning.

Without semantic interoperability, data integration can lead to misinterpretation, introduce bias or noise during training, and ultimately result in inaccurate or non-generalizable model predictions. Achieving semantic interoperability typically requires the adoption of standardized vocabularies and ontologies.

In the context of computer science, ontology is a formal and unambiguous representation of knowledge, expressed in terms of classes, relations, and axiomatic constraints. It provides a structured framework for organizing data, enabling efficient information retrieval, data integration, interoperability, and the discovery of new knowledge.

In materials engineering, ontologies support the development of data models that explicitly define context, including units, relationships, and constraints, as well as mapping and harmonization mechanisms that align heterogeneous schemas within a shared conceptual framework. Artificial intelligence (AI) models trained on semantically interoperable datasets can more readily adapt to new data sources and enable reliable knowledge transfer across platforms.

Despite these advantages, the adoption of semantic technologies in materials engineering remains limited. This is partly due to insufficient expertise in ontology development and the lack of standardized laboratory documentation practices. Manual, unstructured documentation-often created without the use of Electronic Laboratory Notebooks (ELNs)-is still common.

Broadly speaking, two main types of ontologies are distinguished: Domain-Level Ontologies (DLOs) and Top-Level Ontologies (TLOs). DLOs are designed to represent knowledge specific to a particular scientific or technical domain, with structures tailored to domain-specific data. In contrast, TLOs are universal frameworks intended to be applicable across diverse scientific and technological disciplines.

Among the most widely used TLOs in materials science and engineering are Basic Formal Ontology (BFO), Elementary Multiperspective Material Ontology (EMMO), Semanticscience Integrated Ontology (SIO), and Suggested Upper Merged Ontology (SUMO). These ontologies provide conceptual foundations for the development of semantically consistent DLOs. Examples of DLOs applied in materials science include the PMD Core Ontology (PMDco) [339] and the Tensile Test Ontology (TTO) [340]. It should be noted, however, that not all DLOs are formally aligned with a TLO. A comprehensive review of DLOs in materials science was presented by de Baas and co-workers in 2023 [341].

The use of semantic technologies significantly facilitates the preparation of high-quality training datasets for AI models. At the same time, artificial intelligence-particularly Large Language Models (LLMs)-can contribute to Ontology Learning (OL), which is, the semi-automatic or automatic construction of ontologies and semantically interoperable datasets. LLMs are generative models based on Transformer architectures that specialize in processing and generating natural language. A prominent example is ChatGPT.

Most research on OL using LLMs has been published since 2023 [342,343], and to the best of our knowledge, none of these studies has yet focused specifically on materials engineering. Nevertheless, this approach appears highly promising. LLMs can also support the construction of Knowledge Graphs (KGs) and the extraction of structured information from experimental protocols or data sheets [344]. This process, known as Information Extraction (IE), enables the transformation of unstructured text into machine-readable knowledge. A Knowledge Graph is a graph-based representation of real-world facts, typically built upon one or more ontologies, and consists of entities (data instances) and the relationships between them.

Overall, the cited works demonstrate that prompting techniques based on LLMs constitute a promising tool for data standardization and for the development of new semantic technologies applicable to bioactive materials science and engineering. Proper organization and semantic structuring of experimental datasets could substantially enhance knowledge inference and accelerate discovery in this field.

### 5.5 Data, cost, and interpretability: choosing between AI and traditional methods

Advances in AI are making it increasingly difficult to identify scientific or engineering problems for which the recommended solution would not involve at least some element of AI. Nevertheless, there remain many applications where it is worth considering traditional, non-AI methods. Traditional approaches, whether mathematics-based or physics-based, often perform particularly well when the available dataset is small, noisy, or expensive to obtain. In such settings, deep learning methods are difficult to apply effectively, whereas many classical ML techniques (e.g., linear regression, decision trees, support vector machines) can still achieve good accuracy on limited data without significant overfitting.

Another limitation of many AI solutions is their reliance on high-performance hardware such as graphics processing units, tensor processing units, or cloud-based infrastructure. Beyond the need for substantial computing resources, deep learning systems are frequently energy-intensive, making them impractical for certain applications. In contrast, closed-form equations or simple numerical algorithms typically run faster and at lower cost, which is especially advantageous in embedded systems or real-time control scenarios.

Finally, interpretability remains a significant differentiator. Most deep learning models operate as black boxes, providing limited insight into the reasoning behind their predictions and occasionally producing outputs that violate fundamental physical laws (e.g., conservation laws or thermodynamic constraints). By comparison, mathematics-driven methods, such as the finite element method (FEM), offer results that are fully explainable - assuming the problem can be described mathematically - allowing researchers to trace predictions back to well-defined physical principles.

### 5.6 Bridging data scarcity and model overfitting in ML for bioactive materials

One of the main challenges in applying neural networks to the design of bioactive materials is the need for sufficiently large training datasets.

In this context, the main bottleneck is often the laboratory's ability to generate sufficient data within a reasonable timeframe. Various algorithms have been developed to optimize experimental workflows, with active learning being one of the most effective. Nevertheless, obtaining adequate training data remains a non-trivial challenge.

A promising solution is the implementation of self-driving laboratories (SDLs), which automate experimental work through robotics. One notable example is SAMPLE [255], a robotic platform designed to investigate new protein functions by performing biochemical assays. An additional advantage of such systems is their integration with cloud-based infrastructure (self-driving cloud labs), where experiments can be remotely programmed and executed according to user-defined protocols [345]. This approach significantly increases laboratory efficiency and, consequently, provides larger and more reliable datasets for model training and validation.

However, even relatively large datasets do not fully protect against overfitting in neural network-based ML models. Overfitting is one of the most undesirable and difficult-to-eliminate phenomena in ML. Overfitted models capture noise and irrelevant patterns in the data, which leads to poor generalization to new, unseen cases. Various strategies exist to mitigate this problem. A particularly promising approach is the use of physics-informed neural networks (PINNs), which combine data-driven learning with physical modeling [346,347]. Unlike conventional neural networks, PINNs enforce compliance with physical laws expressed through differential equations. Their training relies on minimizing a composite loss function that balances three elements: consistency with experimental data, satisfaction of governing equations, and adherence to boundary or initial conditions.

This framework offers significant advantages. Even with limited data, the network remains constrained by physical principles, reducing the risk of memorizing noise. By embedding physics, PINNs inherently avoid solutions that violate conservation laws, thereby narrowing the space of admissible functions. Furthermore, the use of differential equations can enforce smoothness in the predicted functions, helping to prevent overfitting to spurious fluctuations.

### 5.7 Summary of challenges and opportunities

In summary, the clinical translation of AI-driven bioactive materials is currently limited by multifaceted challenges. Main technical challenges such as overfitting and lack of large, standardized datasets lead to the development of semantic ontologies, active learning, and Physics-Informed Neural Networks (PINNs). Moreover, the epistemological challenges of “black-box” approaches demand the incorporation of Explainable AI (XAI) to ensure true mechanistic understanding. Emerging opportunities for innovation are expected to combine AI with other cutting-edge tools, including digital twins, multi-scale modeling, and synthetic biology, to create smart living materials. However, the realization of this potential is contingent upon the dynamic evolution of existing international regulatory bodies, including the FDA, EMA, MHRA and NMPA, to ensure the safe, transparent, and standardized clinical translation of AI-driven bioactive materials.

## 6 Conclusions

AI is reshaping bioactive materials from trial-and-error experimentation towards data-driven engineering. As introduced in this review, AI technologies have been successfully employed in all spectra of material development pipeline, ranging from inverse design of bioactive materials and optimization of fabrication protocols to prediction of physicochemical properties and assessments of in vitro and in vivo biological processes.

However, to fulfill the clinical translation of AI-driven bioactive materials, several critical challenges and obstacles need to be overcome at the technical, epistemological, and regulatory levels. First, the problem of data availability and heterogeneity requires coordinated efforts in data standardization and the automation of high-throughput experiments using semantic ontologies. Additionally, the limited interpretability of “black-box” deep learning needs to be addressed. Approaches such as XAI and PINNs provide promising solutions to integrate physical laws and mechanistic insight over simple parameter optimization. Furthermore, although some regulatory guidance has been proposed by authorities such as the FDA, EMA, MHRA and NMPA, further evolution and adaptation are still needed to keep pace with the rapid development of AI.

We believe the future of bioactive materials research will be defined by the innovative integration of AI and multidisciplinary technologies, including the elucidation of complex material-cell interactions in vivo, the advancement of automated bioprinting, and the combination of ML and MSB for engineering intelligent and adaptive living materials. Overall, AI has shown enormous potential to accelerate the discovery, design, evaluation, and clinical translation of next-generation bioactive materials.

## CRediT authorship contribution statement

**Jiezhong Shi:** Writing – review & editing, Writing – original draft, Visualization, Funding acquisition, Formal analysis, Data curation, Conceptualization. **Ting Liang:** Writing – review & editing, Writing – original draft, Visualization, Formal analysis, Data curation. **Marcin Heljak:** Writing – review & editing, Writing – original draft, Visualization, Formal analysis, Data curation, Conceptualization. **Zhen Qiu:** Writing – review & editing, Formal analysis, Data curation. **Song Chen:** Writing – review & editing, Formal analysis, Data curation. **Jiaying Li:** Writing – review & editing, Formal analysis, Data curation. **Asimina Kazakidi:** Writing – review & editing, Formal analysis. **Wojciech Święszkowski:** Writing – review & editing, Funding acquisition, Formal analysis, Data curation, Conceptualization. **Bin Li:** Writing – review & editing, Writing – original draft, Supervision, Funding acquisition, Formal analysis, Data curation, Conceptualization. **Wenmiao Shu:** Writing – review & editing, Writing – original draft, Supervision, Funding acquisition, Conceptualization.

## Ethics approval and consent to participate

This article does not involve human participants or animal experiments; therefore, ethical approval and informed consent were not required.

## Declaration of competing interest

The authors declare the following personal relationships which may be considered as potential competing interests: Jiezhong Shi is currently employed by SINOPEC Key Laboratory of Research and Application of Medical and Hygienic Materials, SINOPEC Beijing Research Institute of Chemical Industry Co., Ltd.
